# Re-Du-Ning Alleviates Airway-Intestinal Inflammation of COPD Rats by Regulating Glycerophospholipid Metabolism and Modulating Gut Microbiome

**DOI:** 10.3390/medsci14060662

**Published:** 2026-10-12

**Authors:** Yule Wang, Lanyu Cheng, Chunyan Wang, Xu Wang, Jing Li, Ying Wang, Xiaoyan Gao, Gan Luo

**Affiliations:** 1 School of Chinese Materia Medica, Beijing University of Chinese Medicine, Beijing 100029, China; 20230935137@bucm.edu.cn (Y.W.); 20240935153@bucm.edu.cn (L.C.); 20250935186@bucm.edu.cn (C.W.); wangy174@bucm.edu.cn (Y.W.); gaoxiaoyan@bucm.edu.cn (X.G.); 2 School of Chinese Medicine, Beijing University of Chinese Medicine, Beijing 100029, China; xuwang@bucm.edu.cn (X.W.); lijing131126abc@163.com (J.L.)

**Keywords:** chronic obstructive pulmonary disease, Re-Du-Ning, gut–lung axis, glycerophospholipid metabolism

## Abstract

**Background**: Re-Du-Ning (RDN) is a traditional Chinese medicine (TCM) formulation composed of Lonicera japonica, Gardenia jasminoides, and Artemisia annua. Previous in vitro studies have demonstrated that RDN suppresses pro-inflammatory cytokine release in RAW264.7 macrophages and exhibits potential anti-COPD activity; however, its precise molecular mechanism remains elusive. **Methods**: A rat COPD model was established by combining cigarette smoke exposure and intratracheal instillation of lipopolysaccharide (LPS). Rats were intragastrically administered with gradient doses of RDN to evaluate its in vivo pharmacological efficacy. Untargeted metabolomics and 16S rRNA microbiome sequencing were performed to screen differential metabolites and gut bacterial genera following RDN intervention, followed by Kyoto Encyclopedia of Genes and Genomes (KEGG) enrichment analysis of core metabolic pathways. Further functional validations were conducted via antibiotic-mediated gut microbiota depletion, fecal microbiota transplantation (FMT), targeted glycerophospholipid supplementation, and human clinical cohort datasets from the Gene Expression Omnibus (GEO) to verify the central roles of gut microbiota and glycerophospholipid metabolism. **Results**: RDN intervention partially improved lung-function impairment and attenuated histopathological changes in the lung and colon in rats with COPD, while simultaneously reducing the levels of IL-1β, TNF-α, and IL-6 in serum, BALF, and feces. Mult-omics analysis further revealed that RDN partially restored glycerophospholipid metabolic profiles and reshaped gut microbiota composition, and a “host phenotype–metabolite–gut microbiota” network was constructed using correlation analysis. Finally, antibiotic depletion and faecal microbiota transplantation (FMT) experiments suggested that an intact gut microbiota is required for the full protective effect of RDN and that part of this effect is transferable by FMT; LPC (14:0) and LPC (16:0) can regulate cytokine release in BEAS-2B cells, and data from the GEO human cohort further validated the role of glycerophospholipid metabolism in COPD. **Conclusions**: RDN effectively alleviates symptoms in rats with COPD by regulating the gut microbiota and glycerophospholipid metabolism, providing a comprehensive and in-depth understanding of the multisystem regulatory network underlying COPD.

## 1. Introduction

Chronic obstructive pulmonary disease (COPD) is a heterogeneous pulmonary disorder characterized by progressive decline in lung function and abnormalities in the airways and alveoli. It primarily manifests as chronic respiratory symptoms such as dyspnea, cough, and sputum production, accompanied by persistent airflow limitation. As one of the top three causes of death worldwide [1], COPD has become a major global public health issue. According to statistics, by 2021, the number of deaths had further risen to approximately 3.72 million [2,3]. The situation in China is particularly severe; in 2021, nearly 1.29 million people in China died from COPD, accounting for approximately 35% of the global total [4].

The heavy burden of disease underscores the urgency of COPD prevention and treatment. Factors such as smoking, air pollution, occupational dust exposure, and recurrent respiratory infections can continuously activate the systemic immune system [5,6], leading to chronic inflammatory infiltration of the airways, lung parenchyma, and perivascular spaces [7]. During the inflammatory response, inflammatory cells such as neutrophils and macrophages accumulate in large numbers and become activated, releasing various inflammatory mediators including TNF-α, IL-1β, and IL-6 [5,8,9]. These inflammatory mediators not only directly stimulate the airway mucosa, leading to congestion, edema, and increased exudation, but also amplify the inflammatory response, further recruiting more inflammatory cells to infiltrate the site and creating a persistent inflammatory state [7]. Under the combined influence of the aforementioned inflammatory and immune factors, characteristic pathological changes occur: the airway wall thickens due to inflammatory cell infiltration and collagen deposition, resulting in airflow limitation [10]; submucosal glands and goblet cells in the surface epithelium undergo significant hyperplasia and overexpress mucin, resulting in highly viscous sputum [11]. It is worth noting that, according to the latest update to GOLD 2026, the treatment of COPD cannot focus solely on local airway inflammation; systemic inflammation must also be properly managed [12]. In addition to airway inflammation and emphysema (respiratory symptoms), patients with COPD experience at least one gastrointestinal symptom, such as diarrhea or constipation [13]. Studies have shown that, compared to the general population, the incidence of Crohn’s disease and ulcerative colitis is 55% and 30% higher, respectively, among patients with COPD [14]. In animal models of COPD, rat colonic tissue exhibited severe epithelial damage, disruption of glandular architecture, and inflammatory cell infiltration, with elevated histological scores for colitis and significantly increased levels of inflammatory markers (such as TNF-α, IL-6, and IL-1β) [15].

The “gut–lung” axis is a bidirectional regulatory pathway connecting the gut and the lungs, facilitating cross-organ regulation through interactions among microbiota, metabolites, and the immune system. Mice with COPD exhibit gut microbiota dysbiosis; oral administration of Parabacteroides goldsteinii MTS01 significantly improved their body weight and lung function and reduced the expression of pro-inflammatory cytokines in colonic tissue [16]. Furthermore, the protective effect of the “Nourishing Yin and Clearing the Lungs” decoction in treating pulmonary inflammation is associated with eight key gut bacterial genera, including Prevotellaceae_NK3B31_group. Previous studies have confirmed higher levels of Lachnoclostridium in the gut microbiota of patients with AECOPD [17,18]. In addition to gut microbiota dysbiosis, metabolic profiles in COPD patients exhibit significant differences, particularly in lipid metabolism. Among the top 50 metabolites in fecal samples from COPD patients and healthy individuals, 46% were derived from lipids, suggesting that lipid metabolism may be altered [19]. In the serum of stable-phase COPD patients and healthy smokers, LPC (18:0), LPC (18:1), and LPC (18:2) were significantly negatively correlated with C-reactive protein and IL-6; LPC (18:0) and LPC (18:2) show positive correlations with post-bronchodilator forced expiratory volume in 1 s (FEV_1_), and LPC (18:2) is additionally associated with predicted FEV_1_ percentage, forced vital capacity (FVC) and the FEV_1_/FVC ratio. These findings suggest that dysregulated lysophospholipid metabolism, particularly reduced LPC levels, is causally linked to declined lung function, emphysema progression and interstitial pulmonary lesions [20]. Glycerophospholipids are core components of cell membrane phospholipids; abnormalities in their metabolism can lead to changes in membrane fluidity, disruption of lipid raft structures, and disruption of signal transduction pathways, and promote the arachidonic acid cascade, resulting in the production of potent inflammatory mediators such as prostaglandins and leukotrienes. Collectively, gut microbiota and glycerophospholipid metabolism represent promising therapeutic targets to relieve pulmonary and intestinal inflammation in COPD.

Re-Du-Ning (RDN) is a compound Chinese medicinal formula consisting of three herbal components: *Lonicera japonica*, *Gardenia jasminoides* and *Artemisia annua*. Modern pharmacological investigations have confirmed that flavonoids from *A. annua*, triterpenoid saponins, flavonoids and essential oils from L. japonica, as well as geniposide and dehydroxy geniposide from *G. jasminoides*, exert robust inhibitory effects on pathogenic microbial proliferation and systemic inflammatory responses. Gardenia polysaccharides remodel gut microbiota homeostasis in cholestasis model rats, reduce intestinal LPS translocation into systemic circulation, block the hepatic TLR4/NF-κB inflammatory signaling cascade, and alleviate hepatic inflammatory injuries [21]. Both aqueous extracts of *A. annua* [22] and flower bud polysaccharides of *L. japonica* [23] significantly reduce serum total cholesterol and triglyceride concentrations.

Given the important roles of glycerophospholipid metabolism and the gut microbiota in the pathology and intervention of COPD, we hypothesize that RDN alleviates airway-gut damage in COPD by reshaping the gut microbiota and restoring the homeostasis of glycerophospholipid metabolism. To test this hypothesis, this study used a rat model of COPD induced by smoke exposure combined with intratracheal instillation of lipopolysaccharide (LPS), and administered RDN orally. Using metabolomics and 16S rRNA sequencing techniques, we systematically investigated the potential mechanisms by which RDN alleviates COPD through the regulation of the gut microbiota and lipid metabolism, and validated these findings using fecal microbiota transplantation experiments, targeted glycerophospholipid supplementation, and clinical data from GEO. In summary, this study elucidates the mechanism by which RDN combats COPD from two perspectives: restoring glycerophospholipid metabolic homeostasis and regulating the composition of the gut microbiota. It provides a research model for metabolomics and microbiome studies of the gut–lung axis in COPD and offers a scientific basis for drug development targeting the gut–lung axis in COPD.

## 2. Materials and Methods

### 2.1. Drugs and Reagents

Jiaozi brand cigarettes (containing 11 mg of tar, 1.1 mg of nicotine, and 12 mg of nitric oxide per cigarette) were purchased from Sichuan China Tobacco Industry Co., Ltd.(Chengdu, Sichuan province, China); Re-Du-Ning Injection (National Drug Approval No. Z 20050217, Lot No.: 210711) was purchased from Jiangsu Kangyuan Pharmaceutical Co., Ltd.(Lianyungang, Jiangsu province, China); Lipopolysaccharide (Lot No.: L2800) was purchased from Sigma-Aldrich (St. Louis, MO, USA); Rat IL-6 Assay Kit (Lot No.: KT3066-A), IL-1β Assay Kit (Lot No.: KT2923-A), TNF-α Assay Kit (Lot No.: KT3056-A), and LPS Assay Kit (Lot No.: MM-0647R1) were purchased from Jiangsu Kete Biotechnology Co., Ltd.(Yancheng, Jiangsu province, China); Dexamethasone Acetate Tablets (National Drug Approval No. H33020833, Lot No.: LB2495) were purchased from Zhejiang Xianju Pharmaceutical Co., Ltd.(Taizhou, Zhejiang province, China); neomycin sulfate (Lot No.: S17028), vancomycin (Lot No.: Y25829), and metronidazole (Lot No.: S17079) were purchased from Shanghai Yuanye Biotechnology Co., Ltd.(Shanghai, China); and ampicillin (Lot No.: A830931) was purchased from Shanghai McLean Biochemical Technology Co., Ltd(Shanghai, China).

### 2.2. Preparation of RDN Gastric Administration Solution

The Re-Du-Ning Injection was refrigerated at −80 °C to pre-freeze it, then lyophilized. Different doses of RDN lyophilized powder were weighed and reconstituted with sterile PBS to prepare the RDN administration solution for later use.

To anchor the animal doses to clinical use, the human daily dose was converted to a rat-equivalent dose on the basis of geniposide content. This human geniposide dose was converted to rat equivalent by body-surface-area normalisation using the standard correction factors (human Km = 37, rat Km = 6; conversion factor ≈ 6.3). The three doses were approximately 1×, 5× and 10× the clinical geniposide equivalent, with the lowest dose serving as the clinical reference.

### 2.3. Animal Grouping and Drug Administration

**Figure 1:**
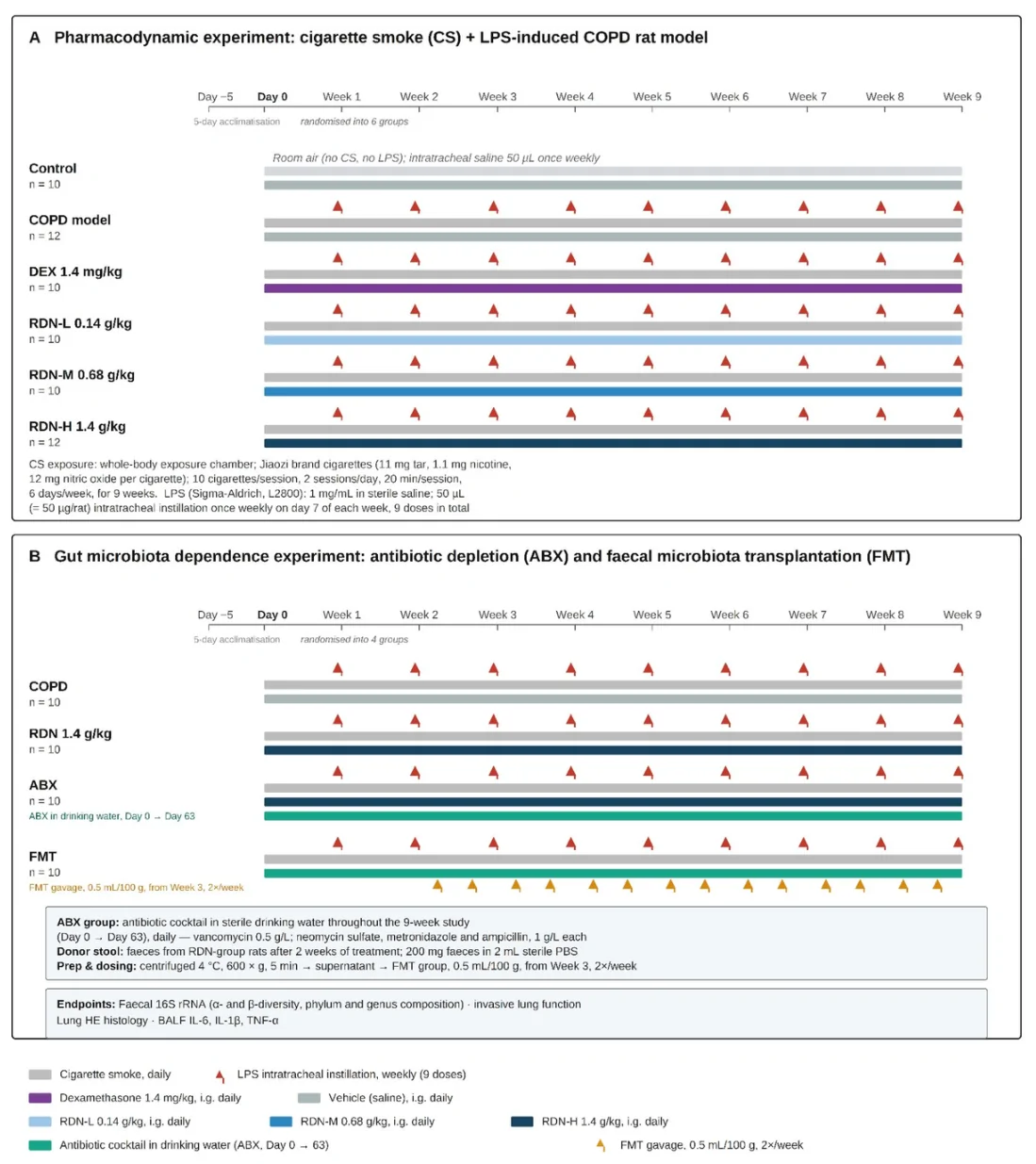
Schematic of the experimental design. (**A**) Pharmacodynamic study. Male Wistar rats (*n* = 64) were randomised to six groups after a 5-day acclimatisation: Control (room air + intratracheal saline), COPD model, dexamethasone (DEX, 1.4 mg/kg), and RDN-L/M/H (0.14, 0.68, 1.4 g/kg). (**B**) Gut microbiota-dependence study. Male Wistar rats (*n* = 40) received the identical CS + LPS regimen and were allocated to four groups (COPD; RDN; ABX; FMT).

Male Wistar rats (6 weeks old, 200 g, *n* = 64) purchased from Sibeifu (Beijing) Biotechnology Co., Ltd(Beijing, China). were used in the pharmacological efficacy experiments. All experimental animals were housed in the containment system at Beijing University of Chinese Medicine under constant temperature conditions with a 12-h light–dark cycle, an ambient temperature of 20 ± 2 °C, and relative humidity of 40–70%, with free access to food and water. Rats were exposed to cigarette smoke in a whole-body exposure chamber using Jiaozi brand cigarettes (11 mg tar, 1.1 mg nicotine, 12 mg nitric oxide per cigarette) at 10 cigarettes per session, 2 sessions per day, for 20 min per session, 6 days per week, for 9 consecutive weeks. LPS (Sigma-Aldrich, L2800) was dissolved in sterile saline at 1000 µg per 1 mL. For each administration, 50 μL of this LPS stock solution was administered via intratracheal instillation once weekly on day 7 of each week, for a total of 9 doses. Control rats were handled identically but exposed to room air and received intratracheal instillation of 50 μL sterile saline. Body weight and general condition were monitored weekly throughout the 9-week period. After a 5-day acclimatization period, the animals were randomly divided into 6 groups: a control group, a COPD group, a dexamethasone (DEX, 1.4 mg/kg) group, and low-, medium-, and high-dose RDN groups (0.14, 0.68, 1.4 g/kg). The control group was administered saline via gavage, while the model group was exposed to daily smoke and received weekly intratracheal LPS instillation. The positive control group was administered dexamethasone via gavage, and the high-, medium-, and low-dose RDN groups were administered the corresponding doses of RDN via gavage. The gavage volume for each rat was 0.1 g/mL (Figure 1A).

For the antibiotic and fecal microbiota transplantation experiments, male Wistar rats (6 weeks old, 200 g, *n* = 40) were purchased from Sbeifu (Beijing) Biotechnology Co., Ltd(Beijing, China). The housing and care conditions for the experimental animals were the same as those in the pharmacodynamic experiments. After a 5-day acclimatization period, the rats were randomly divided into four groups: the COPD group, the RDN (RDN, 1.4 g/kg) group, the antibiotic (ABX) group, and the fecal microbiota transplantation (FMT) group. All rats were exposed to smoke daily and received weekly intratracheal LPS instillation. On this basis, each group received the following treatments: the COPD group was gavaged with saline daily, while the RDN and ABX groups were gavaged with RDN. Concurrently, the ABX and FMT groups were provided with sterile water containing a mixture of antibiotics for daily drinking. The mixture consisted of vancomycin at 0.5 g/L, neomycin sulfate, metronidazole, and ampicillin, each at 1 g/L. After 2 weeks of continuous treatment, feces were collected from the RDN group rats, resuspended in sterile PBS, and diluted at a ratio of 2 mL PBS per 200 mg of feces. The mixture was thoroughly mixed until no large particles were visible. The resuspension was centrifuged at 4 °C and 600 g for 5 min, and the supernatant was collected as the fecal microbiota suspension. Subsequently, this suspension was administered via gavage to rats in the FMT group as a single dose of 0.5 mL per 100 g of body weight (Figure 1B).

Rats were randomly allocated into different experimental groups using a random number table before modeling. Investigators were not blinded during animal handling and sample collection. All animal experimental procedures in this study were reviewed and approved by the Ethics Committee for Animal Care and Treatment at Beijing University of Chinese Medicine (approval number: BUCM-2025021402-1041; approval date: 14 February 2025 and approval number: BUCM20250907-008; approval date: 7 September 2025). Animals were handled in strict accordance with the institutional guidelines for laboratory animal welfare.

### 2.4. Sample Collection

On day 63 of the intervention, fresh fecal samples were collected from the rats and frozen in liquid nitrogen. The rats were anesthetized via intraperitoneal injection of 1.5% sodium pentobarbital (50 mg/kg), intubated, and their lung function was measured using a pulmonary function testing system. Blood was then drawn from the abdominal aorta and centrifuged at 3000 r/min at 4 °C for 10 min to separate the serum, which was set aside. The thoracic cavity was opened to expose the trachea and lung tissue. The right pulmonary hilum was ligated, and 2 mL of phosphate-buffered saline (PBS) was drawn into a syringe, injected into the left lung tissue, and then aspirated back to collect the lavage fluid. This procedure was repeated three times, and the lavage fluids were combined. Finally, the right lung was removed, blood was rinsed off with saline, surface moisture was blotted away, and the right middle lobe was fixed in 4% paraformaldehyde; the remainder was stored in cryovials at −80 °C for later use. A 5-cm section of distal colon tissue was taken, blood and colonic contents were rinsed away with saline, surface moisture was blotted off, a portion of the colon tissue was fixed in 4% paraformaldehyde, and the remainder was stored in a cryovial at −80 °C for later use in subsequent testing.

### 2.5. Measurement of Inflammatory Cytokines in BALF, Serum, and Feces

The instructions in the ELISA kit manual were strictly followed to measure the expression levels of TNF-α, IL-6, and IL-1β in BALF, serum, and fecal samples, respectively.

### 2.6. Hematoxylin and Eosin (HE) Staining

After fixing rat right lung and colon tissues in 4% paraformaldehyde for 48 h, the tissues were dehydrated, routinely embedded in paraffin, and sectioned into 5-μm-thick sections, which were then stained with hematoxylin and eosin (HE). Pathological changes were observed under a light microscope, and images were captured.

### 2.7. Immunohistochemistry

After paraffin embedding of the fixed colon tissue, sections were cut to a thickness of 4–5 μm using a microtome. The sections were sequentially placed in Eco-Friendly Dewaxing Solution I for 10 min, Eco-Friendly Dewaxing Solution II for 10 min, Eco-Friendly Dewaxing Solution III for 10 min, Anhydrous Ethanol I for 5 min, Anhydrous Ethanol II for 5 min, and Anhydrous Ethanol III for 5 min, and finally washed with distilled water. Antigen retrieval was performed on the sections using sodium citrate buffer (pH 6.0) in a microwave oven for 10 min. Samples were washed three times with PBS, blocked with endogenous peroxidase with H_2_O_2_, and blocked with goat serum at room temperature for 30 min. The primary antibodies (Anti-ZO-1 and Anti-Occludin) were added dropwise and incubated overnight at 4 °C. Goat anti-rabbit secondary antibody was added dropwise and incubated at room temperature for 1 h. After washing three times with PBS, the sample was developed with DAB for 5–10 min and then counterstained with hematoxylin for 10 s. Finally, it was mounted with neutral resin and observed under a microscope, and images were captured. ZO-1 and occludin proteins appear brown. Semi-quantitative analysis was performed using grayscale analysis with ImageJ 1.52a software.

### 2.8. Non-Targeted Metabolomics Analysis

Untargeted metabolomic analysis was performed on lung tissue, serum, and fecal samples from the control group, model group, and high-dose RDN group.

Chromatographic conditions: A Thermo Scientific Vanquish separation system (Thermo Fisher Scientific(Waltham, MA, USA)) was used with an ACQUITY UPLC HSS T3 column (2.1 mm × 100 mm, 1.8 μm). The injection volume was 2 μL, with the column temperature and sample chamber temperature set at 35 °C and 4 °C, respectively. The mobile phase consisted of 0.1% formic acid in water (A) and acetonitrile (B), with the following optimized gradient: 0–2 min, 2–30% B; 2–6 min, 30–70% B; 6–10 min, 70–98% B; 10–13 min, 98% B; 13–13.1 min, 98–2% B; 13.1–16 min, 2% B, with a flow rate of 0.3 mL/min.

Mass spectrometry conditions: A Thermo Q Exactive Plus high-resolution mass spectrometry system (Thermo Fisher Scientific(Waltham, MA, USA)) was used, with an electrospray ion source. The spray voltages in positive and negative ion modes were 4.2 kV and −3.5 kV, respectively. Data were acquired in both positive and negative ion modes using data-dependent acquisition (DDA) mode, with a scan range of m/z 150–1500, a capillary temperature of 320 °C, a sheath gas flow rate of 35 arb, an auxiliary gas flow rate of 15 arb, an S-lens voltage set to 50 V, and MS and MS/MS resolutions of 70,000 and 17,500, respectively.

Unsupervised principal component analysis (PCA) was used to assess the degree of separation among the three sample groups. Components showing significant intergroup differences were screened based on the criteria of Variable Importance in Projection (VIP) > 1 and *p* < 0.05. Metabolites were identified through comparison with the HMDB and Lipid databases, based on fragmentation data, exact mass, and retention time of the features. To identify metabolic pathways associated with RDN-induced remission of COPD, the screened differentially expressed metabolites were entered into the MetaboAnalyst 6.0 website (https://www.metaboanalyst.ca/) (accessed on 1 March 2026) for pathway analysis.

### 2.9. Gut Microbiome Analysis

Fecal samples from rats in the control group, model group, and high-dose RDN group were selected for 16S rRNA sequencing analysis. Quality control of the raw paired-end sequencing reads was performed using the fastp software (version 0.19.6), and sequence assembly was conducted using FLASH (version 1.2.11). Sequences that passed quality control and assembly were clustered into operational taxonomic units (OTUs) based on 97% similarity, and chimeric sequences were excluded. The RDP Classifier (version 2.11) [24] was used to align the sequences against the Silva 16S rRNA gene database (v138) for OTU taxonomic annotation, with a confidence threshold set at 70%. Community composition was analyzed at each taxonomic level. PICRUSt2 (version 2.2.0) was used for 16S functional prediction analysis. Abundance data from the 18 samples were normalized to a uniform level of 35,516 sequences per sample, resulting in a total of 2230 OTUs.

Both the α-diversity index and β-diversity analysis were calculated using the Mothur software (version 1.30.2) and visualized using R (version 3.3.1). Principal Coordinate Analysis (PCoA) based on Bray–Curtis distance, combined with the ANOSIM/Adonis method, was used to assess overall differences in bacterial communities among different samples. Community bar charts were used to visually illustrate the distribution of dominant species. The Kruskal–Wallis test (multiple group comparison) in R was used to assess significant differences between groups. Furthermore, LEfSe analysis was employed to identify species that significantly contributed to the differences between groups. All data analyses were performed on the Meiji Bio Cloud Platform (https://v.majorbio.com).

### 2.10. In Vitro Cell Assay

BEAS-2B cells were seeded at a density of 1 × 10^5^ cells per well in a 6-well plate. After the cells adhered, the culture medium was discarded and the wells were washed with PBS. The cells were divided into a control group, a model group, and a treatment group. The control and model groups were treated with standard culture medium, while the treatment group was treated with a solution containing 10 μM LPC. After 2 h of culture, 2 μg/mL of LPS was added to the model and treatment groups. Following an additional 24 h of culture, the cell supernatants were collected and centrifuged, and the levels of IL-6, TNF-α, and IL-1β were measured in each group.

### 2.11. GEO Cohort Validation

Sequencing datasets were retrieved from the NCBI Gene Expression Omnibus (GEO) database using the keyword “COPD”. Differential expression analysis of the selected datasets was performed using GEO2R. Genes satisfying the criteria of |log2FC| > 0.58 and adjusted *p*-value < 0.05 were defined as differentially expressed genes.

### 2.12. Data Analysis

Statistical analysis of the collected data was performed using SPSS 23.0 software. Normality (Shapiro–Wilk test) and homogeneity of variance (Levene’s test) were assessed prior to all comparisons. For comparisons among three or more groups, one-way ANOVA was applied if both assumptions were satisfied, followed by Tukey’s HSD post hoc test. When the assumptions of normality or homogeneity of variance were violated, nonparametric tests were used instead of parametric ANOVA. Results are expressed as mean ± SD, and *p* < 0.05 indicates a statistically significant difference; GraphPad Prism 9 software was used for data analysis and visualization; the Maiwei Cloud platform was used to perform Spearman’s correlation analysis on host phenotypes, differentially expressed metabolites, and differentially expressed microbial communities, calculating the correlation coefficient (r) and its significance, and plotting correlation heatmaps based on the correlation coefficients. Cytoscape 3.10.0 was used for visual network analysis. A “host phenotype–metabolite–microbiota” interaction network was constructed with host phenotypes, differentially expressed metabolites, and differentially expressed microbiota as nodes and significant correlations as edges.

## 3. Results

### 3.1. RDN Alleviated Lung Injury in Rats with COPD

**Figure 2:**
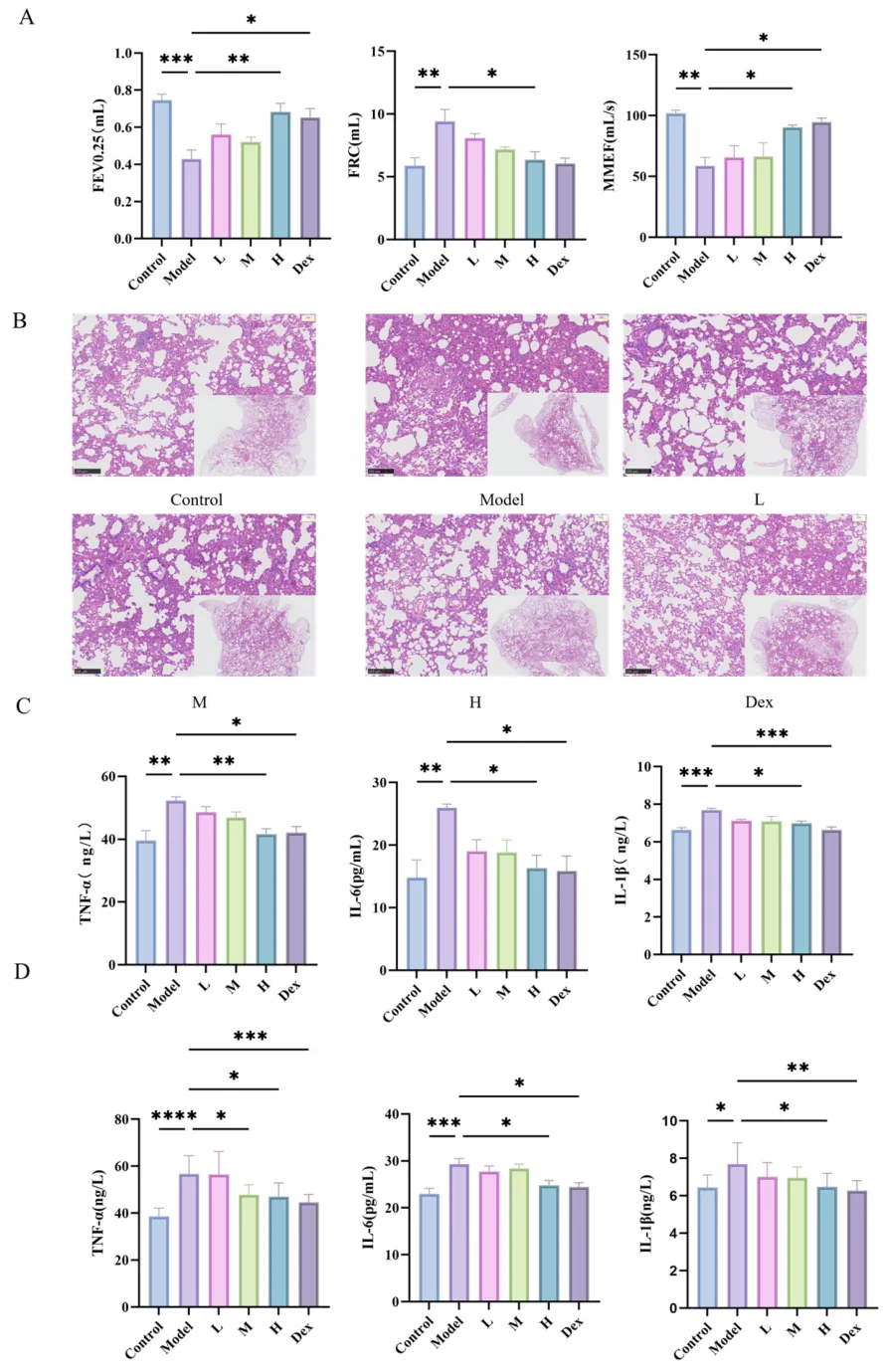
RDN alleviates lung injury in rats with COPD: (**A**) Changes in invasive respiratory function parameters (FEV_0.25_, FRC, and MMEF) in each group (*n* = 6); (**B**) Histopathological changes in lung tissue in each group (HE staining, magnification ×100) (*n* = 3); (**C**) Expression of inflammatory cytokines TNF-α, IL-6, and IL-1β in BALF; (**D**) Expression of inflammatory factors TNF-α, IL-6, and IL-1β in serum (*n* = 6) (compared with the model group, * *p* < 0.05, ** *p* < 0.01, *** *p* < 0.001, **** *p* < 0.0001).

Pulmonary function test results showed that, compared with the control group, the COPD group exhibited significantly reduced forced expiratory volume in 0.25 s (FEV_0.25_) and mean mid-expiratory flow (MMEF), as well as increased functional residual capacity, indicating severe impairment of pulmonary function in the COPD group; these abnormalities were alleviated following RDN intervention (Figure 2A). HE staining revealed severe pathological changes in lung tissue, including ruptured alveolar walls and massive infiltration of inflammatory cells; these histological alterations were attenuated by RDN in a dose-dependent manner. Alveolar architecture remained partially distorted even at the highest dose and did not fully return to that of control lungs (Figure 2B). To further clarify the effects of RDN on inflammatory changes in COPD, we measured the levels of inflammatory cytokines in BALF and serum from rats in each group. As shown in Figure 2D and Figure 2C, levels of IL-6, IL-1β, and TNF-α in BALF and serum were significantly elevated in the COPD group; RDN downregulated the expression of these pro-inflammatory factors in a dose-dependent manner, thereby alleviating pulmonary inflammation.

### 3.2. RDN Improves Intestinal Injury and Barrier Dysfunction in Rats with COPD

**Figure 3:**
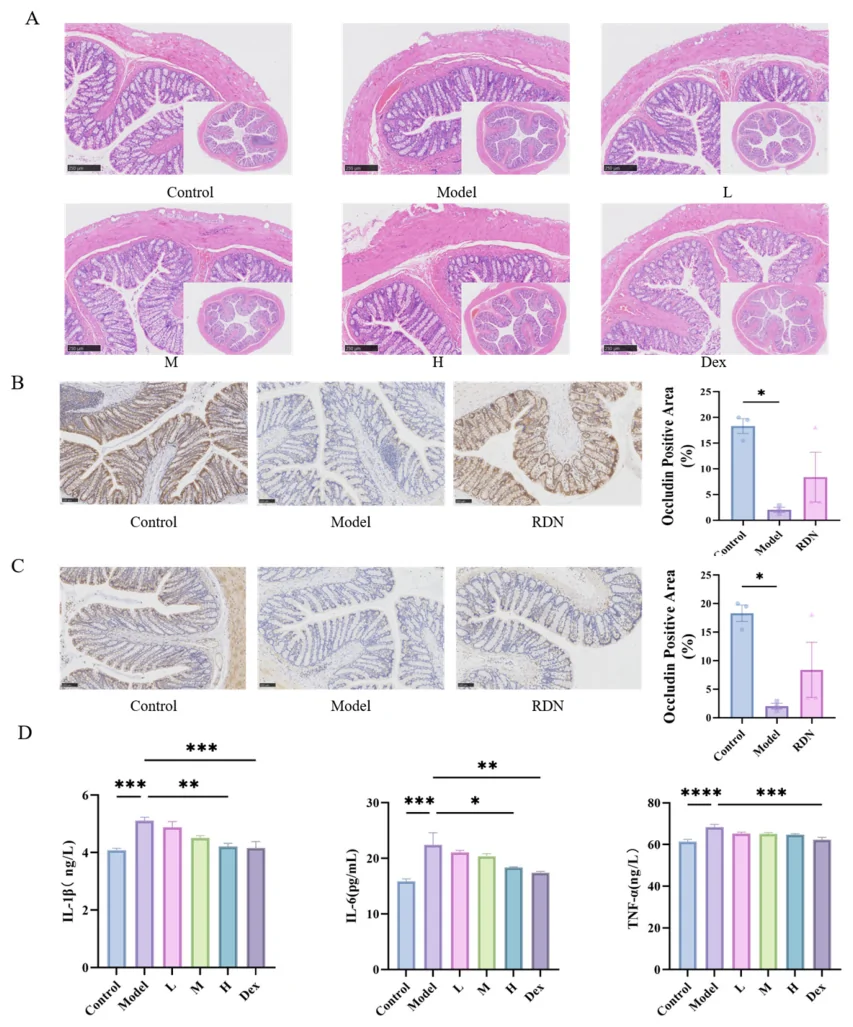
RDN alleviates intestinal mucosal damage and barrier dysfunction in rats with COPD. (**A**) Representative images of H&E staining in colon tissue sections (*n* = 3); (**B**) Representative images and quantitative analysis of Occludin expression by IHC in colon tissue (*n* = 3); (**C**) Representative images and quantitative analysis of ZO-1 expression by IHC in colon tissue (*n* = 3); (**D**) Levels of TNF-α, IL-6, and IL-1β in feces (*n* = 6) (compared with the model group, * *p* < 0.05, ** *p* < 0.01, *** *p* < 0.001, **** *p* < 0.0001).

As shown in Figure 3A, compared with the control group, the colons of COPD rats exhibited significant inflammatory cell infiltration, whereas intestinal inflammation was alleviated following RDN intervention. ZO-1 and Occludin are markers of colonic tight junctions; immunohistochemical analysis was performed to assess the expression levels of these two proteins in the colons of rats from the three groups. As shown in Figure 3B,C, ZO-1 and Occludin were stained brownish-black. In the colonic tissue of the Control group, tight proteins were uniformly distributed across the surface of intestinal mucosal cells. Compared with the Control group, the ZO-1 and Occludin-positive areas in the colonic tissue of COPD rats were reduced, indicating a decrease in the levels of intestinal tight proteins in the COPD group, suggesting impaired intestinal barrier function and disruption of intestinal structure. In the RDN-H group, the positive areas for both proteins were increased relative to the COPD group but remained below control values, indicating partial rather than complete restoration of tight-junction protein expression. Additionally, it was found that levels of TNF-α, IL-6, and IL-1β were significantly elevated in the feces of COPD rats, whereas RDN downregulated the expression of these three inflammatory factors in a dose-dependent manner.

### 3.3. RDN Partially Restores Glycerophospholipid Metabolic Homeostasis in Rats with COPD

**Figure 4:**
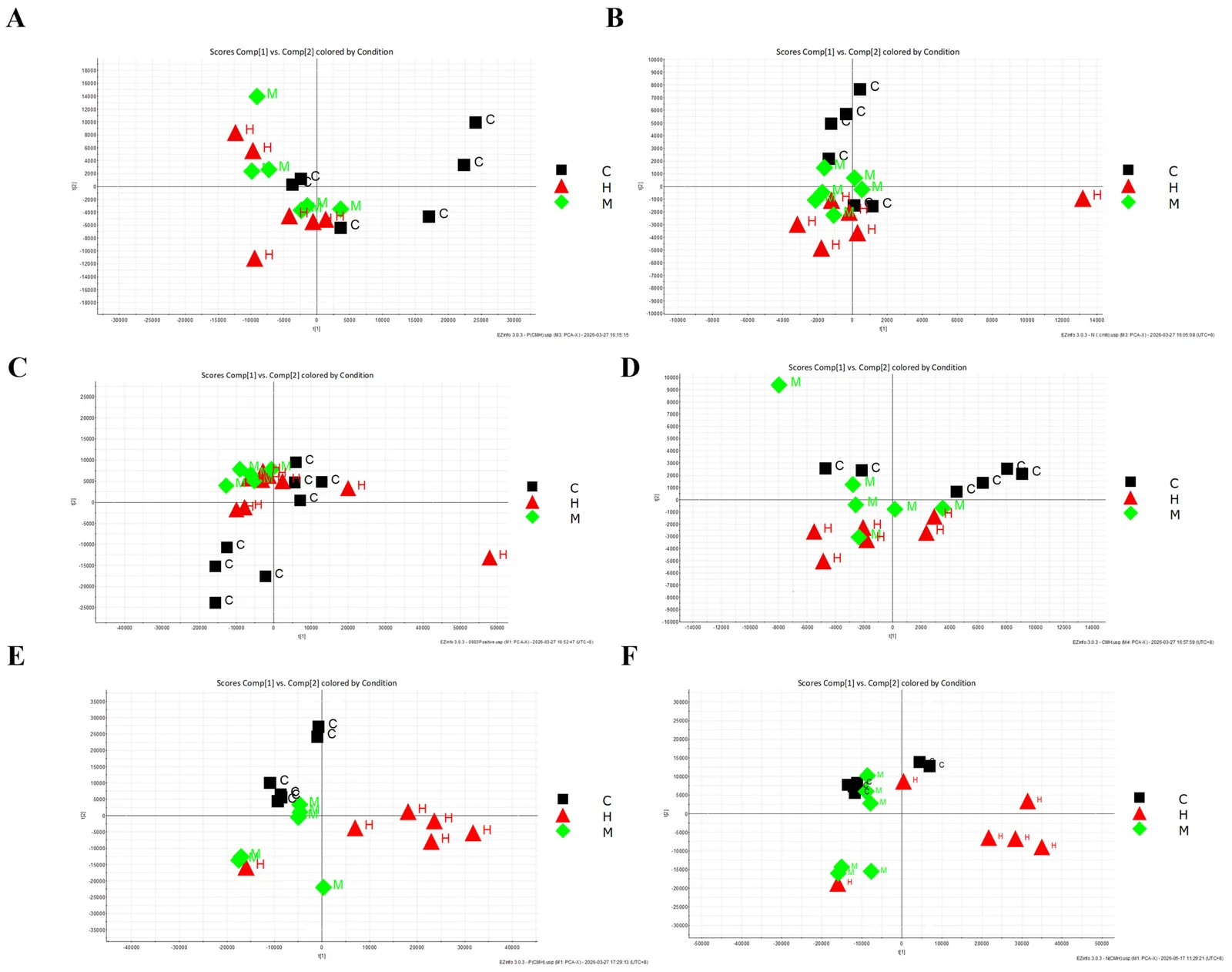
PCA score plots of metabolic profiles in RDN-treated COPD rats. (**A**) PCA score plot of lung tissue across different groups in cation mode; (**B**) PCA score plot of lung tissue across different groups in anion mode; (**C**) PCA score plot of serum samples across different groups in cation mode; (**D**) PCA score plot of serum samples across different groups in anion mode; (**E**) PCA score plot of fecal samples across different groups in cation mode; (**F**) PCA score plot of fecal samples across different groups in anion mode. C, control group; M, COPD group; H, RDN treatment group.

To clarify the differences in metabolites between the COPD model and rats treated with RDN, we performed non-targeted metabolomics analysis on lung tissue, serum, and fecal samples. The PCA score plot showed that the metabolic profiles of lung tissue, serum, and fecal samples from the three groups of rats were distributed in distinct regions (Figure 4A–F). Using VIP > 1 and *p* < 0.05 as screening criteria to identify differentially expressed metabolites among the three groups. The results showed that, compared with the Control group, 47 metabolites in lung tissue from the COPD group exhibited changes in both anionic and cationic modes; of these, 5 were significantly downregulated by RDN (details are given in Supplementary Table S1.), including PS (18:0/18:1 (9Z)), PC (18:0/0:0), ADP, PA (16:0/14:0), and NADPH. In serum, 92 metabolites were altered in the COPD group compared to the Control group under both positive and negative ion modes, of which 21 were significantly modulated by RDN (details are given in Supplementary Table S2), including deoxycholic acid, NAD, sphinganine 1-phosphate, and CDP-glucose. In fecal samples, 238 metabolites showed changes in the COPD group compared to the Control group under both positive and negative ion modes, of which 40 were significantly modulated by RDN (details are given in Supplementary Table S3), including 7-ketodeoxycholic acid, chenodeoxycholic acid, coprocholic acid, cholic acid, deoxycholic acid, and deoxycholic acid glycine conjugate.

**Figure 5:**
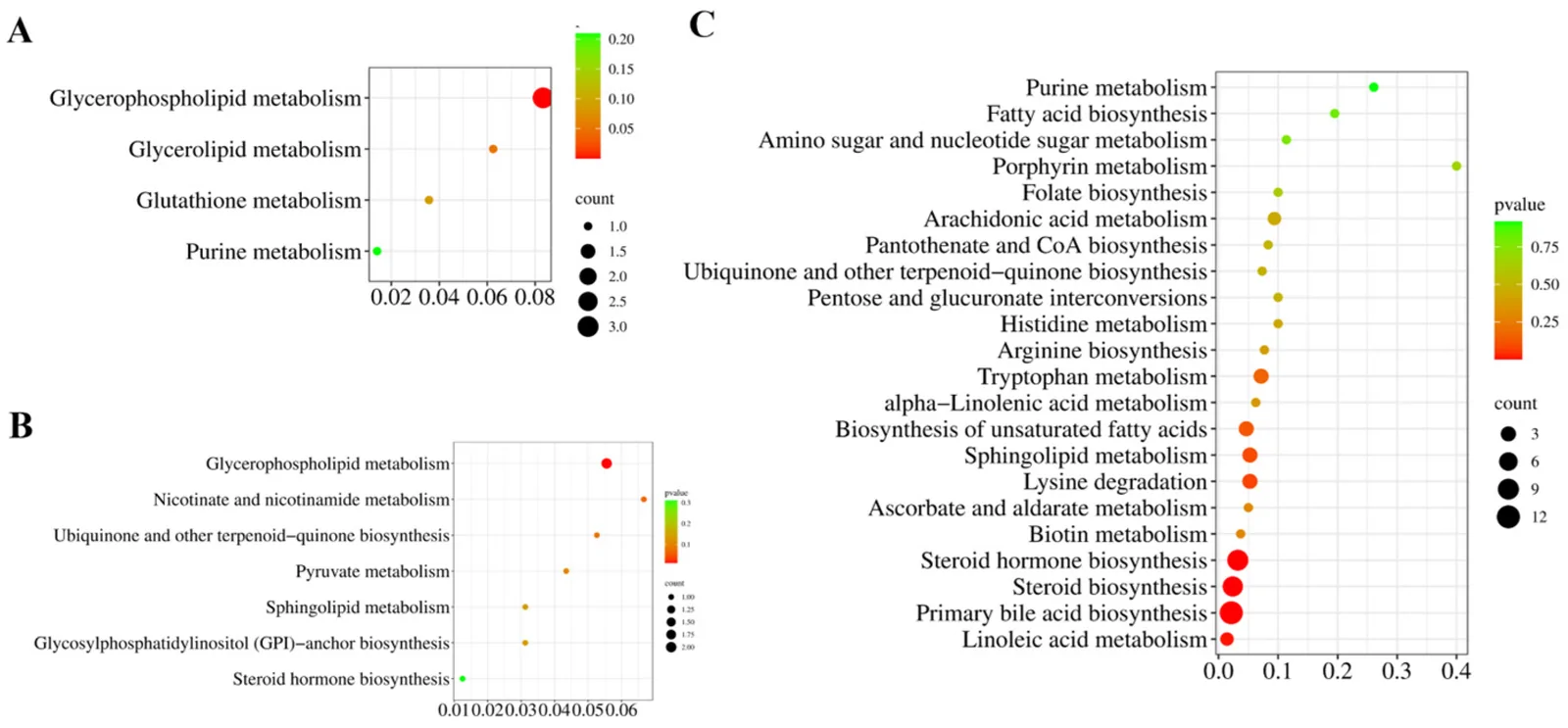
KEGG pathway enrichment bubble plots for differentially expressed metabolites across sample groups. (**A**) KEGG enrichment of differentially expressed metabolites in lung tissue of the RDN reversal group; (**B**) KEGG enrichment of differentially expressed metabolites in serum of the RDN reversal group; (**C**) KEGG enrichment of differentially expressed metabolites in fecal samples of the RDN reversal group.

To investigate how RDN alleviates COPD by regulating metabolism in the lungs, blood, and feces, we performed pathway analysis of differentially expressed metabolites in various biological samples using MetaboAnalyst 6.0. The results showed that in lung tissue, RDN regulates glycerophospholipid metabolism, glyceride metabolism, glutathione metabolism, and purine metabolism (Figure 5A); in serum, RDN modulates metabolic pathways including glycerophospholipid metabolism, nicotinic acid and nicotinamide metabolism, the biosynthesis of ubiquinone and other terpenoquinones, sphingolipid metabolism, and steroid hormone biosynthesis (Figure 5B); and in feces, RDN regulates metabolic pathways including primary bile acid biosynthesis, the biosynthesis of ubiquinone and other terpenoquinones, the biosynthesis of unsaturated fatty acids, steroid biosynthesis, arachidonic acid metabolism, fatty acid biosynthesis, and steroid hormone biosynthesis (Figure 5C).

### 3.4. Re-Shaping of the Gut Microbiome Composition in Rats with COPD by RDN

**Figure 6:**
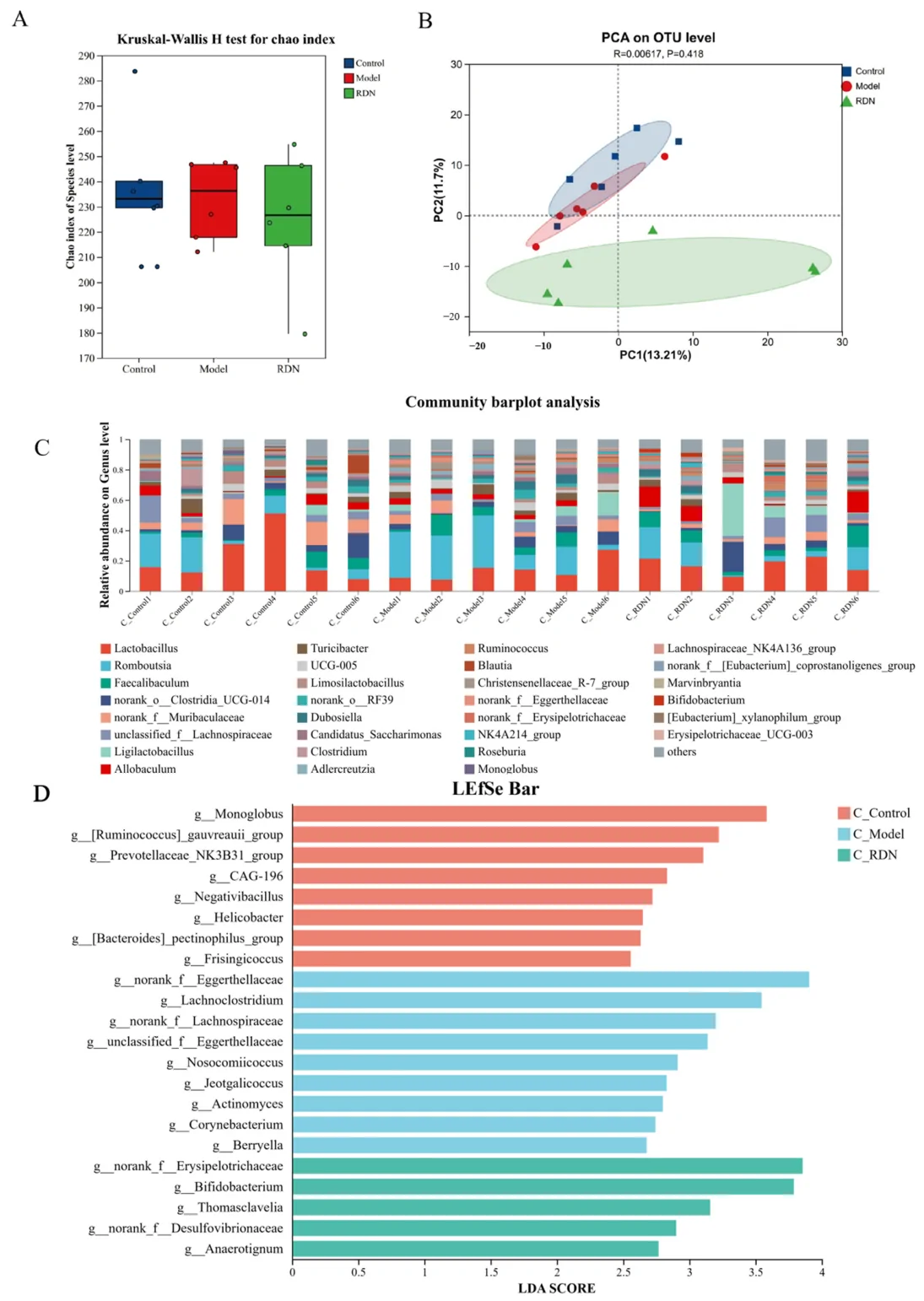
Effects of RDN intervention on gut microbial diversity and community composition in COPD rats. (**A**) Chao1 index at the OTU level; (**B**) PCA at the OTU level; (**C**) Genus-level composition of the microbiota in each group; (**D**) Linear Discriminant Analysis (LDA) scores. Control, control group; Model, COPD group; RDN, RDN treatment group.

The Chao-1 index indicated differences in species richness of the gut microbiota among the groups (Figure 6A); PCA revealed differences in gut microbiota composition at the OTU level among the groups (Figure 6B). Analysis of species composition revealed that the gut microbiota of rats in the COPD group was altered at the genus level, and RDN intervention could reshape the gut microbiota structure in COPD rats (Figure 6C). LEfSe analysis showed that in COPD rats, *g_Actinomyces*, *g_Berryella*, *g_Jeotgalicoccus*, *g_Lachnoclostridium*, *g_Nosocomiicoccus* and *g_unclassified_f_Eggerthellaceae* were the dominant genera; after RDN administration, *g_unclassified_f_Prevotellaceae* and *g_Bifidobacterium* became the dominant genera. These findings indicate that the gut microbiota composition was imbalanced in all three groups of rats, and that RDN can modulate the gut microbiota composition in rats with COPD.

### 3.5. Correlation Analysis Reveals That RDN Alleviates COPD by Regulating the Gut Microbiome and Glycerophospholipid Metabolism

**Figure 7:**
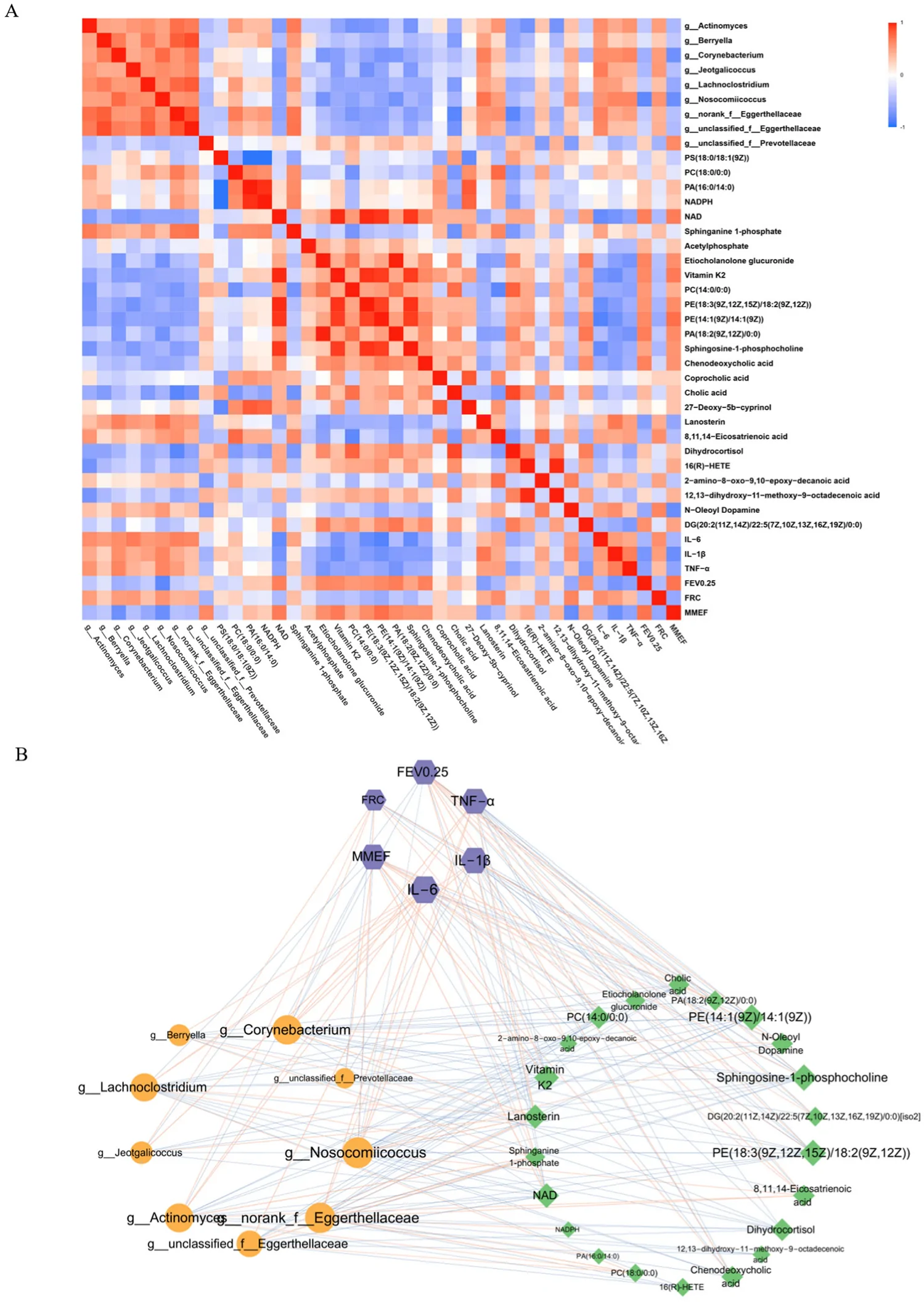
Association analysis of differential microbiota, metabolites, and inflammatory phenotype indicators. (**A**) Heatmap showing the correlation coefficients among differential microbiota, metabolites, and inflammatory phenotype indicators. Red represents positive correlation, and blue represents negative correlation. (**B**) Correlation network of differential microbiota, metabolites, and inflammatory phenotype indicators. The lines indicate significant correlations, with red lines for positive correlations and blue lines for negative correlations.

To further investigate the mechanism by which RDN alleviates COPD, we used Spearman’s correlation analysis to construct a correlation heatmap and a network diagram. Glycerophospholipid metabolism is the central mediating pathway linking the gut microbiota and inflammatory factors in this study. As shown in Figure 7, the levels of various glycerophospholipid metabolites (including lysophosphatidylcholine LPC (14:0), phosphatidylethanolamine PE (18:3/18:2), lysophosphatidic acid LPA (18:2), and diglycerides DG (20:2/22:5)) were negatively correlated with the abundances of *g_Lachnoclostridium*, *g_Corynebacterium*, and *g_Nosocomococcus* (*p* < 0.05) and positively correlated with the abundance of *Prevotellaceae.* They were negatively correlated with the levels of key inflammatory factors such as IL-6, IL-1β, and TNF-α, while being positively correlated with MMEF and FEV_0.25_ and negatively correlated with FRC. These results suggest that RDN can alleviate COPD by regulating the gut microbiota and glycerophospholipid metabolic pathways.

### 3.6. An Intact Gut Microbiota Is Required for, and Partially Transfers, the Protective Effect of RDN

**Figure 8:**
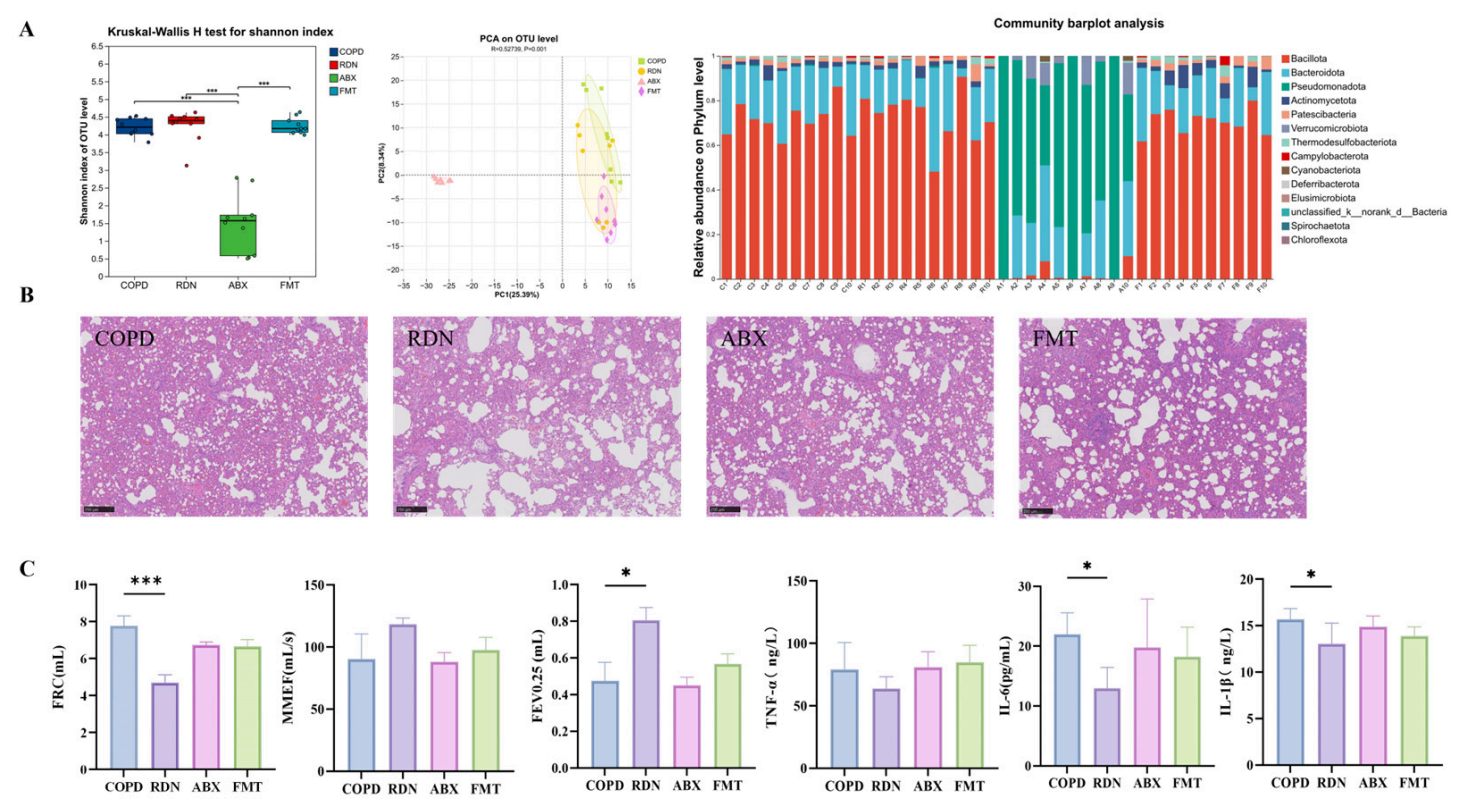
Results of gut microbiota characteristics, lung histopathology, inflammatory cytokines, and pulmonary function parameters in rats across different groups. (**A**) Shannon diversity index and PCA scores at the OTU level, as well as the microbial community composition at the phylum and genus levels for each group (*n* = 10); (**B**) Histopathological changes in lung tissue from each group (HE staining, magnification ×100) (*n* = 3); (**C**) Expression of inflammatory cytokines TNF-α, IL-6, and IL-1β in BALF; changes in invasive respiratory function parameters (FEV_0.25_, FRC, and MMEF) across groups (*n* = 6) (compared with the COPD group, * *p* < 0.05, *** *p* < 0.001).

To clarify the role of the gut microbiota in RDN’s alleviation of COPD, we investigated changes in RDN’s efficacy following the depletion of the gut microbiota via antibiotic treatment and after fecal microbiota transplantation (FMT), to test whether an intact gut microbiota is required for the protective effect of RDN and whether this effect can be transferred by the microbiota itself. As shown in Figure 8A, compared with the RDN group, rats in the ABX group exhibited reduced α-diversity (Shannon index) of the gut microbiota, as well as decreased evenness and richness of microbial composition, whereas the FMT group showed a trend toward recovery. β diversity analysis showed that the FMT group’s microbial community structure was more similar to that of the RDN group, whereas the ABX group differed significantly from both groups, suggesting that after antibiotics eliminated the gut microbiota, the regulatory effect of RDN on the microbiota was blocked. At the phylum level, the RDN group and the FMT group were dominated by Bacteroidetes and Firmicutes, whereas the ABX group showed a significant reduction in Firmicutes abundance, with Proteobacteria becoming the predominant phylum. At the genus level, *Klebsiella* was significantly enriched in the ABX group, while the FMT group showed a trend toward normalization. This suggests that after antibiotics eliminated the microbiota, the role of RDN in regulating microbiota structure was weakened, whereas fecal microbiota transplantation (FMT) led to a certain degree of recovery in gut microbiota structure. Compared with the RDN group, rats in the ABX group did not show relief from inflammatory cell infiltration in lung tissue or improvement in lung function, indicating that an intact gut microbiota is required for the full protective effect of RDN. Following oral administration of RDN-containing feces, compared to the COPD group, the rats in the FMT group showed reduced inflammatory infiltration on lung histology, partial improvement in lung-function parameters, and lower BALF concentrations of TNF-α, IL-6 and IL-1β. These results indicate that RDN-associated microbiota transplantation partially reproduced the benefits of RDN on lung histology, lung function and BALF cytokine concentrations. Taken together, the depletion and transfer experiments support a contributory role for the gut microbiota in the effect of RDN; they do not establish a complete causal chain running from RDN to specific taxa, through defined metabolites, to the lung phenotype.

### 3.7. LPC Species Differentially Modulate Cytokine Release in COPD: Roles in Airway Inflammation and Glycerophospholipid Metabolism

**Figure 9:**
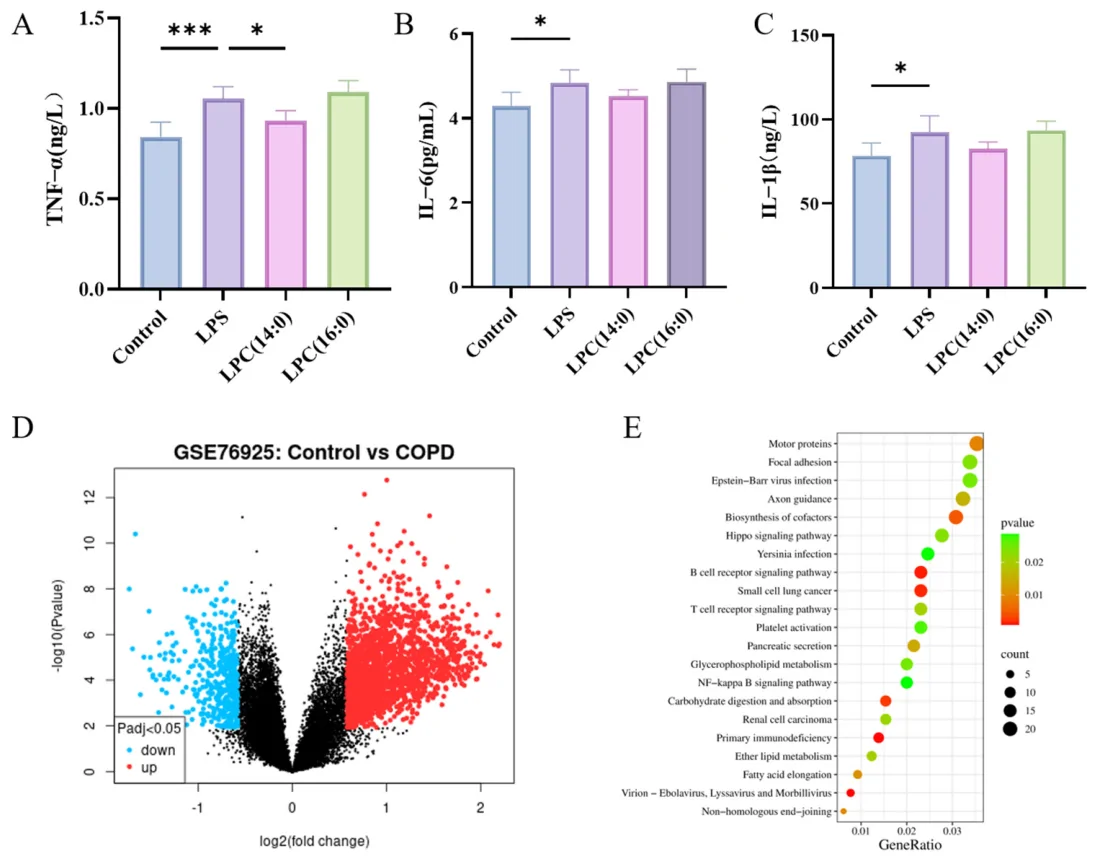
LPC-induced release of inflammatory factors and enrichment analysis of differentially expressed genes in COPD. (**A**) TNF-α expression levels; (**B**): IL-6 expression levels; (**C**): IL-1β expression levels; (**D**) Volcano plot of differentially expressed genes, Volcano plot of differentially expressed genes, where red dots represent significantly upregulated genes, blue dots represent significantly downregulated genes, and black dots represent genes with no significant difference. (**E**): KEGG pathway enrichment bubble plot. Compared with the LPS group, * *p* < 0.05, *** *p* < 0.001.

To determine the effects of LPC (14:0) and LPC (16:0) on the release of inflammatory factors from LPS-induced BEAS-2B cells, a Control group, an LPS group, and an LPC treatment group were established to observe the effects of LPC on the cells. As shown in Figure 9A–C, following LPS treatment, IL-6 levels were significantly elevated in the LPS group. After treating the cells with solutions of different metabolites, LPC (14:0) treatment reduced IL-6, IL-1β, and TNF-α, while LPC (16:0) treatment increased IL-6, IL-1β, and TNF-α. The results indicate that LPC (14:0) tends to inhibit the release of inflammatory factors in LPS-induced BEAS-2B cells, whereas LPC (16:0) tends to promote the release of inflammatory factors. Further differential gene KEGG enrichment analysis was performed using the GEO database GSE76925 (111 COPD lung tissue samples and 40 controls). As shown in Figure 9D,E, the glycerophospholipid metabolism pathway was found to be disrupted; genes disrupted in this pathway included *LCLAT1*, *PLA2G12A*, *PLA2G4A*, *PLA2G2D*, and *PLA2G2A*.

## 4. Discussion

Pulmonary function testing is the clinical gold standard for COPD diagnosis, where post-bronchodilator FEV_1_/FVC < 0.7 defines persistent airflow limitation. In rodent COPD models, FEV_0.25_ is adopted for lung function measurement. Rats have rapid respiration, and their whole forced expiration completes within 0.2–0.5 s; FEV_0.25_ reliably reflects the early rapid expiratory phase. MMEF evaluates mid-expiratory airflow and acts as a marker for small airway obstruction, the primary pathological lesion of COPD. Elevated FRC indicates air trapping due to reduced lung elastic recoil, equivalent to pulmonary hyperinflation detected by body plethysmography in patients. Prior work demonstrated increased TLC and RV, alongside decreased VC, FVC, FEV_0.25_ and dynamic compliance in COPD rats [25]. Consistent with those findings, our model group showed reduced FEV_0.25_ and MMEF and higher FRC versus controls. All RDN doses partially restored these parameters (increased FEV_0.25_ and MMEF, decreased FRC) without full recovery to control values, showing incomplete relief of airway obstruction. COPD lung dysfunction arises from irreversible airflow limitation driven by airway remodeling and loss of lung parenchymal elasticity, accompanied by impaired gas exchange. Alveolar fusion reduces elastic recoil [26] and elevates residual volume; thickened airway walls, narrowed lumens and mucus plugs increase airway resistance and lower the FEV_1_/FVC ratio. Healthy lung tissue presents minimal inflammatory infiltration and unobstructed airways, whereas COPD airway epithelium displays prominent pathological alterations with abundant inflammatory cells in the submucosa and peribronchial regions. In our model rats, alveolar fusion and extensive inflammatory infiltration were observed. RDN treatment attenuated pulmonary inflammatory infiltration relative to the model group, demonstrating its protective effects against COPD-associated lung pathology. Such lesions originate from sustained chronic inflammation. COPD is widely recognized as an inflammatory airway disease: risk factors including smoking and air pollution injure airway epithelium, activate innate and adaptive immunity, recruit inflammatory cells and trigger release of mediators such as TNF-α, IL-1β and IL-6 [27]. These cytokines directly damage lung tissue and promote mucus overproduction and airway smooth muscle hyperplasia, with oxidative stress further aggravating injury. Exaggerated pulmonary inflammatory immune responses represent the core pathological hallmark of COPD [28]. In line with this, BALF IL-6, IL-1β and TNF-α concentrations were markedly higher in the model group than controls, confirming robust airway inflammation in our COPD rat model.

The functional coupling of the lung and large intestine derives from traditional Chinese medicine, with the Huangdi Neijing stating that “the lung connects with the large intestine”. A landmark 2014 study demonstrated that pulmonary inflammation can arise from the intestine, establishing the gut–lung axis as a promising research and therapeutic target for COPD. The gut–lung axis refers to bidirectional crosstalk between the respiratory tract and intestine mediated by microbiota, metabolites, and immune and neural systems [29]. Clinically, a large proportion of COPD patients present gastrointestinal comorbidities [30,31]. Compared with the general population, COPD patients show a 40–70% higher risk of IBD or IBS, and this risk positively correlates with COPD severity [32,33,34]. Animal COPD models also display severe intestinal pathological changes, including disrupted intestinal structure, mucosal thinning, crypt atrophy and distortion, and substantial inflammatory infiltration, indicating intestinal barrier injury [35,36,37]. Consistent with these reports, our COPD rats exhibited obvious colonic villus atrophy and inflammatory infiltration, reduced expression of tight-junction proteins Occludin and ZO-1, and increased TNF-α, IL-6 and IL-1β in serum and feces. These data suggest that, besides pulmonary inflammatory injury, marked intestinal damage also occurs in COPD rats. The GOLD 2026 guidelines emphasize the importance of managing systemic inflammation in COPD, and multiple studies support gut–lung-axis-targeted interventions for COPD [38,39,40]. For example, probiotic *Pediococcus* SMM914 reshapes gut microbiota, increases short-chain fatty acid and antioxidant metabolite production, and relieves lung injury in murine COPD [41]. Compound CKDB315 elevates fecal *Lactiplantibacillus* and short-chain fatty acids and improves respiratory symptoms in patients [42]. In our study, RDN treatment decreased IL-6, IL-1β and TNF-α levels in BALF, serum and feces, demonstrating that RDN ameliorates airway and intestinal inflammation in COPD.

We further explored key mechanistic questions: whether gut microbiota mediate the anti-COPD effects of RDN and which metabolites participate in gut–lung crosstalk. Antibiotic depletion experiments (Figure 8) showed that the protective effects of RDN against COPD-related airway inflammation were attenuated under antibiotic-induced pseudo-sterile conditions, demonstrating that intestinal bacteria are necessary to achieve the full therapeutic efficacy of RDN in this model. Moreover, FMT using fecal microbiota from RDN-treated rats partially recapitulated the therapeutic benefits, indicating that a portion of RDN’s protective effects can be transferred via RDN-modulated gut microbiota. We performed 16S rRNA sequencing to compare bacterial genera between control and COPD rats, as well as between COPD and RDN-treated rats. β-diversity analysis revealed distinct genus-level microbial compositions across the control, COPD and RDN groups. LEfSe identified *g_Actinomyces*, *g_Berryella*, *g_Jeotgalicoccus*, *g_Lachnoclostridium*, *g_Nosocomiicoccus*, and *g_unclassified_f_Eggerthellaceae* as signature genera in the COPD model. After RDN treatment, *g_unclassified_f_Prevotellaceae* and *g_Bifidobacterium* became dominant. Consistent with prior gut–lung-axis studies [43], *Lachnoclostridium*, which has been reported to be enriched in AECOPD patients [17,18], was abundant in our COPD rats, while *g_unclassified_f_Prevotellaceae* and *Bifidobacterium* bloomed following RDN intervention.

We propose that glycerophospholipids act as key metabolites mediating gut–lung crosstalk. Progressive loss of lung function is a hallmark of COPD, which arises from structural remodeling of alveoli, the fundamental respiratory units. Alveoli are composed of alveolar membranes and surface surfactant, where glycerophospholipids represent core constituents. Substantial shifts in glycerophospholipid profiles alter alveolar biophysical properties and eventually impair respiratory function. Cumulative clinical evidence indicates that dysregulated glycerophospholipid metabolism is tightly linked to COPD onset, disease severity and acute exacerbations [44,45,46,47]. Individual glycerophospholipid species may serve as candidate COPD biomarkers and correlate with inflammatory cytokines and lung-function parameters [20,48,49,50]. As the gut–lung axis supports bidirectional lung–intestinal communication, our multi-omics data suggest that pathological disruption of pulmonary and circulating glycerophospholipid metabolism triggers intestinal metabolic disorders and gut injury. RDN restores glycerophospholipid profiles in feces and serum, which in turn rescues glycerophospholipid homeostasis in lung tissue and mitigates airway inflammation. Accordingly, glycerophospholipid metabolism constitutes a critical pathway connecting the gut–lung network. Previous studies have demonstrated that *Prevotella* [51] and *Bifidobacterium* [52,53] are closely associated with glycerophospholipid metabolism. UniProt database queries reveal that these two genera encode key enzymes for glycerophospholipid biosynthesis, providing a plausible mechanistic explanation for the metabolic recovery induced by RDN-reshaped gut microbiota. Building upon these in vivo and microbiome findings, our in vitro experiments in BEAS-2B cells further demonstrated opposing inflammatory regulatory activities of distinct LPC species. LPC (14:0) reduced LPS-induced secretion of IL-6, IL-1β and TNF-α, while LPC (16:0) further elevated these pro-inflammatory cytokines. These findings indicate that individual LPC isoforms exert divergent effects on airway epithelial inflammation, which may partially account for the complex shifts in glycerophospholipid metabolism observed in our COPD rat model. Consistent with these cellular observations, KEGG enrichment analysis based on the human COPD transcriptomic dataset GSE76925 further supported the involvement of glycerophospholipid metabolism. Multiple key genes governing LPC metabolism (*LCLAT1*, *PLA2G12A*, *PLA2G4A*, *PLA2G2D*, and *PLA2G2A*) were dysregulated in COPD lung tissues. Collectively, these data suggest that disrupted glycerophospholipid metabolism may contribute to COPD airway inflammation by altering the balance between anti-inflammatory and pro-inflammatory LPC species.

More broadly, these findings fit within growing research on microbiome-targeted pharmacology for chronic inflammatory disorders. Cytokine-blocking biologics can secondarily reshape microbial communities: a recent systematic review of IL−4/IL−13 blockade with dupilumab in type 2 inflammatory diseases reported variable shifts in airway and gut microbiota alongside clinical improvement, showing microbiome changes may be both a cause and consequence of immune intervention [54]. In respiratory disease, COPD airway microbiota vary by disease severity, exacerbation frequency and inhaled corticosteroid use [55]. Collectively, these data suggest the gut–lung axis is pharmacologically tractable. Our results raise unresolved questions: whether RDN-enriched taxa (*Prevotellaceae*, *Bifidobacterium*) drive protection or merely serve as biomarkers, and whether the LPC-dominated glycerophospholipid signature mediates inflammation reduction or only reflects it.

The cigarette smoke (CS) plus lipopolysaccharide (LPS) rat COPD model is widely adopted and recapitulates key human pathological features: persistent airflow limitation (reduced FEV_0.25_ and MMEF, elevated FRC), emphysematous alveolar destruction, sustained inflammatory infiltration, and, as shown here, intestinal barrier injury with increased systemic and luminal cytokines. Weekly intratracheal LPS instillation mimics recurrent respiratory infections that trigger acute exacerbations and accelerate disease progression, a feature poorly reproduced by CS exposure alone. Nevertheless, several limitations restrict direct clinical extrapolation. (i) Heterogeneity: human COPD presents diverse phenotypes including small-airway disease, different emphysema subtypes, chronic bronchitis and extrapulmonary comorbidities with multiple inflammatory endotypes. Inbred Wistar rats receiving uniform CS/LPS treatment develop a relatively homogeneous phenotype and cannot recapitulate this clinical diversity. (ii) Chronicity: human COPD develops over decades of exposure, while our 9-week protocol induces early-to-intermediate pulmonary remodeling instead of fully established disease. (iii) Lung function readouts: rodent FEV0.25 is a surrogate index and not equivalent to clinical post-bronchodilator FEV_1_. We did not evaluate bronchodilator reversibility, diffusing capacity, exacerbation frequency, symptom scores or exercise tolerance. (iv) Drug administration: RDN was delivered via gavage in rats, whereas clinical administration is intravenous (Section 2.2), leading to incomparable systemic exposure. Within these constraints, mechanistic findings are more translatable than quantitative outcomes. First, concurrent airway injury, gut dysbiosis and intestinal barrier dysfunction mirror gastrointestinal comorbidity in COPD patients [13,14,30]. Second, correlations between glycerophospholipids (especially LPCs), lung function and inflammatory cytokines align with clinical lipidomic reports linking circulating LPCs and FEV_1_ [20]. Third, analysis of human lung transcriptome dataset GSE76925 identified perturbed glycerophospholipid metabolism in COPD, confirming this pathway’s relevance beyond rodent models. Thus, our data provide mechanistic insights and candidate biomarkers (gut microbial taxa and LPC-centered metabolic signature) to power future clinical studies rather than prove RDN’s clinical efficacy.

Additional limitations affect causal interpretation of our results. First, microbiota experiments demonstrated microbial requirement and partial transferable protection but not a complete causal cascade. Antibiotic depletion is non-specific and alters intestinal metabolites, immunity and drug absorption; our FMT experiment lacked stool donors from untreated COPD or vehicle control rats. Therefore, we cannot attribute transferred therapeutic effects to the LEfSe-identified taxa, and the causal relationship between microbial composition and glycerophospholipid profiles remains unclear. Second, metabolomic analysis was performed only in the high-dose RDN group; dose-dependent metabolic changes were not assessed. Metabolite annotation relied on accurate mass, retention time and MS/MS matching without authentic standards, so LPC identifications are putative. Third, the mechanistic exploration described in Section 3.7 was conducted in cultured BEAS-2B bronchial epithelial cells in vitro. The cellular intervention setting cannot fully recapitulate the complex in vivo microenvironment of animal COPD models; since we did not validate these molecular mechanisms in animal tissues, these data only indicate the trend and specificity of LPC effects rather than physiological relevance. Future studies are warranted to verify these mechanisms in animal samples. Finally, human validation relies solely on one public lung transcriptomic dataset, supporting pathway-level changes without clinical metabolomic or microbiome evidence.

## 5. Conclusions

In rats with a smoke-and-LPS-induced COPD model, inflammatory damage was observed in the lungs and colon, accompanied by dysregulation of glycerophospholipid metabolism in the lungs, blood, and feces, as well as disruption of the fecal microbiota structure. RDN partially restored glycerophospholipid metabolic homeostasis in lung, blood and faeces and reshaped gut microbiota composition, accompanied by attenuation—but not resolution—of lung and intestinal injury.

## Data Availability

The original contributions presented in the study are included in the article and Supplementary Materials. Further inquiries can be directed to the corresponding author.

## Supplementary Materials

The following supporting information can be downloaded at: https://www.mdpi.com/article/10.3390/medsci14060662/s1: Table S1: Identification of differential metabolites in lung; Table S2: Identification of differential metabolites in serum; Table S3: Identification of differential metabolites in feces.

## Abbreviations

The following abbreviations are used in this manuscript:

ABX	Antibiotics
ANOVA	Analysis of variance
BALF	Bronchoalveolar Lavage Fluid
COPD	Chronic obstructive pulmonary disease
DEX	Dexamethasone
ELISA	Enzyme-linked immunosorbent assay
FEV_0.25_	Forced expiratory volume in 0.25 s
FMT	Fecal Microbiota Transplantation
FRC	Functional Residual Capacity
FVC	Forced vital capacity
GEO	Gene Expression Omnibus
GOLD	Global Initiative for Chronic Obstructive Lung Disease
HE	Hematoxylin and eosin staining
IL-6	Interleukin-6
IL-1β	Interleukin-1β
KEGG	Kyoto Encyclopedia of Genes and Genomes
LEfSe	Linear discriminant analysis Effect Size
LPA	Lysophosphatidic Acid
LPC	Lysophosphatidylcholine
LPS	lipopolysaccharide
MMEF	Maximal Mid-Expiratory Flow
NF-κB	Nuclear factor-κB
OTU	Operational Taxonomic Unit
PCA	Principal component analysis
PE	Phosphatidylethanolamine
RDN	Re-Du-Ning
RV	Residual volume
TCM	Traditional Chinese Medicine
TLC	Total lung capacity
TNF-α	Tumor necrosis factor-α;
VC	Vital capacity

## References

[1] Xiao X., Guo W., Ding Z., Chen N., Zhang Q. (2025). Mechanistic insights into baicalein’s anti-inflammatory effects in COPD: Targeting oxidative stress and CD8^+^ T cell cytotoxicity. Phytomedicine.

[2] Wang Z., Lin J., Liang L., Huang F., Yao X., Peng K., Gao Y., Zheng J. (2025). Global, regional, and national burden of chronic obstructive pulmonary disease and its attributable risk factors from 1990 to 2021: An analysis for the Global Burden of Disease Study 2021. Respir. Res..

[3] Naghavi M., Kyu H.H., Aalipour M.A., Aalruz H., Ababneh H.S., Abafita B.J., Abaraogu U.O., Abbafati C., Abbasi M., Abbaspour F. (2025). Global burden of 292 causes of death in 204 countries and territories and 660 subnational locations, 1990–2023: A systematic analysis for the Global Burden of Disease Study 2023. Lancet.

[4] Primary COPD Prevention and Management Office of Basic Public Health Service Project, National Office for Chronic Respiratory Disease Prevention and Control, National Center for Respiratory Medicine, China Japan Friendship Hospital, Expert Committee on Primary COPD Prevention and Treatment, Respiratory Disease Prevention and Control Committee of Chinese Preventive Medicine Association, General Practice Branch of Chinese Medical Association, Compilation Expert Group of National Implementation Guidelines for Prevention and Management of Chronic Obstructive Pulmonary Disease at Primary Care Level (2026). National Implementation Guidelines for Prevention and Management of Chronic Obstructive Pulmonary Disease at Primary Care Level (2025). Chin. J. Gen. Pract..

[5] Hikichi M., Mizumura K., Maruoka S., Gon Y. (2019). Pathogenesis of chronic obstructive pulmonary disease (COPD) induced by cigarette smoke. J. Thorac. Dis..

[6] Upadhyay P., Wu C.-W., Pham A., Zeki A., Royer C., Kodavanti U., Takeuchi M., Bayram H., Pinkerton K. (2023). Animal models and mechanisms of tobacco smoke-induced chronic obstructive pulmonary disease (COPD). J. Toxicol. Environ. Health B.

[7] Bracke K., Brusselle G. (2025). Update on the pathogenetic hallmarks of chronic obstructive pulmonary disease. Presse Méd..

[8] Cheng D.Y. (2015). Efficacy Analysis of Traditional Chinese Medicine in Inhibiting Inflammatory Response of Chronic Obstructive Pulmonary Disease Based on Modern Literatures. Master’s Thesis.

[9] Liu C.B. (2018). The Role and Mechanism of IL-37 in Cigarette Smoke-Induced Pulmonary Inflammation in Mice. Master’s Thesis.

[10] Polosukhin V.V., Gutor S.S., Du R.H., Richmond B.W., Massion P.P., Wu P., Cates J.M., Sandler K.L., Rennard S.I., Blackwell T.S. (2021). Small airway determinants of airflow limitation in chronic obstructive pulmonary disease. Thorax.

[11] Hill D.B., Button B., Rubinstein M., Boucher R.C. (2022). Physiology and pathophysiology of human airway mucus. Physiol. Rev..

[12] Global Initiative for Chronic Obstructive Lung Disease (2026). Global Strategy for the Diagnosis, Management, and Prevention of Chronic Obstructive Pulmonary Disease: 2026 Report.

[13] Peerlings D.E.M., Sprooten R.T.M., Wouters E.F.M., Lenaerts K., Reynaert N.L., Groenen M.T.J., Houben-Wilke S., Simons S.O., Franssen F.M.E. (2026). Gut integrity, systemic inflammation and exacerbations of COPD: The role of I-FABP. Respir. Med..

[14] Brassard P., Vutcovici M., Ernst P., Patenaude V., Sewitch M., Suissa S., Bitton A. (2015). Increased incidence of inflammatory bowel disease in Québec residents with airway diseases. Eur. Respir. J..

[15] Tao L., Zhang Q., Liu L., Wang K., Wang J., Liu X., Zhao P., Li J. (2024). Inhibition of AhR disrupts intestinal epithelial barrier and induces intestinal injury by activating NF-κB in COPD. FASEB J..

[16] Lai H.-C., Lin T.-L., Chen T.-W., Kuo Y.-L., Chang C.-J., Wu T.-R., Shu C.-C., Tsai Y.-H., Swift S., Lu C.-C. (2022). Gut microbiota modulates COPD pathogenesis: Role of anti-inflammatory *Parabacteroides goldsteinii* lipopolysaccharide. Gut.

[17] Yan J., Wu Z., Deng L., Huang C., Jing Y., Chen X.Y., Xu Y. (2024). Comprehensive analysis of the gut microbiota in patients with chronic obstructive pulmonary disease of varying severity-A prospective, observational study. Heliyon.

[18] Wu Y., Luo Z., Liu C. (2021). Variations in fecal microbial profiles of acute exacerbations and stable chronic obstructive pulmonary disease. Life Sci..

[19] Bowerman K.L., Rehman S.F., Vaughan A., Lachner N., Budden K.F., Kim R.Y., Wood D.L.A., Gellatly S.L., Shukla S.D., Wood L.G. (2020). Disease-associated gut microbiome and metabolome changes in patients with chronic obstructive pulmonary disease. Nat. Commun..

[20] Zhou Q., Xing L., Ma M., Qiongda B., Li D., Wang P., Chen Y., Liang Y., ChuTso M., Sun Y. (2025). Lysophospholipid metabolism, clinical characteristics, and artificial intelligence-based quantitative assessments of chest CT in patients with stable COPD and healthy smokers. Sci. Rep..

[21] Lv C., Liu X., Chen S., Yi Y., Wen X., Li T., Qin S. (2024). Extract of *Gardenia jasminoides* Ellis Attenuates High-Fat Diet-Induced Glycolipid Metabolism Disorder in Rats by Targeting Gut Microbiota and TLR4/Myd88/NF-κB Pathway. Antioxidants.

[22] Park S.-J., Choi C., Jeong J., Lee R.-K., Hwang Y. (2020). *Artemisia annua* extract ameliorates high-fat diet-induced fatty liver by activating AMPK. J. Basic Transl. Res..

[23] Wang D., Zhao X., Liu Y.-L. (2017). Hypoglycemic and hypolipidemic effects of a polysaccharide from flower buds of *Lonicera japonica* in streptozotocin-induced diabetic rats. Int. J. Biol. Macromol..

[24] Stackebrandt E., Goebel B.M. (1994). Taxonomic Note: A Place for DNA-DNA Reassociation and 16S rRNA Sequence Analysis in the Present Species Definition in Bacteriology. Int. J. Syst. Bacteriol..

[25] Chen P.R., Cui L., Hou J.-F. (2007). Research Progress on Animal Models of Chronic Obstructive Pulmonary Disease. Acta Lab. Anim. Sci. Sin..

[26] Rodrigues S.O., Cunha C., Soares G.M.V., Silva P.L., Silva A.R., Gonçalves-de-Albuquerque C.F. (2021). Mechanisms, Pathophysiology and Currently Proposed Treatments of Chronic Obstructive Pulmonary Disease. Pharmaceuticals.

[27] Hogea P., Tudorache E., Fira-Mladinescu O., Marc M., Velescu D., Manolescu D., Bratosin F., Rosca O., Mavrea A., Oancea C. (2023). Serum and Bronchoalveolar Lavage Fluid Levels of Cytokines in Patients with Lung Cancer and Chronic Lung Disease: A Prospective Comparative Study. J. Pers. Med..

[28] Wang J., Ren C., Jin L., Batu W. (2023). Seabuckthorn Wuwei Pulvis attenuates chronic obstructive pulmonary disease in rat through gut microbiota-short chain fatty acids axis. J. Ethnopharmacol..

[29] Wang L., Cai Y., Garssen J., Henricks P.A.J., Folkerts G., Braber S. (2023). The Bidirectional Gut–Lung Axis in Chronic Obstructive Pulmonary Disease. Am. J. Respir. Crit. Care Med..

[30] Rutten E.P.A., Lenaerts K., Buurman W.A., Wouters E.F.M. (2014). Disturbed intestinal integrity in patients with COPD: Effects of activities of daily living. Chest.

[31] Kirschner S.K., Deutz N.E.P., Jonker R., Olde Damink S.W.M., Harrykissoon R.I., Zachria A.J., Dasarathy S., Engelen M.P.K.J. (2021). Intestinal function is impaired in patients with Chronic Obstructive Pulmonary Disease. Clin. Nutr..

[32] Shen Z., Qiu B., Chen L., Zhang Y. (2023). Common gastrointestinal diseases and chronic obstructive pulmonary disease risk: A bidirectional Mendelian randomization analysis. Front. Genet..

[33] Ellrichmann M., Bethge J., Boesenkoetter J., Conrad C., Noth R., Bahmer T., Nikolaus S., Aden K., Zeissig S., Schreiber S. (2021). Subclinical Pulmonary Involvement in Active IBD Responds to Biologic Therapy. J. Crohns Colitis.

[34] Jacobsen H.A., Karachalia Sandri A., Weinreich U.M., Jess T., Larsen L. (2024). Increased risk of obstructive lung disease in inflammatory bowel disease: A population-based cohort study. UEG J..

[35] Liu X., Ding R., Wu K., Zhang A., Fan H., Yang J., Li M. (2025). Exploring the effects of lactulose on lung-intestinal tissue-associated factors and the TLR4/NF-κB signaling pathway in COPD rats based on lung-gut axis theory. Microb. Pathog..

[36] Wang P., Tao W., Li Q., Ma W., Jia W., Kang Y. (2025). Indole-3-Aldehyde alleviates lung inflammation in COPD through activating Aryl Hydrocarbon Receptor to inhibit HDACs/NF-κB/NLRP3 signaling pathways. J. Mol. Med..

[37] Özçam M., Lynch S.V. (2024). The gut–airway microbiome axis in health and respiratory diseases. Nat. Rev. Microbiol..

[38] Muhammad Younus K. (2022). Effect of Probiotic Oral Suspension on Disease Severity in Chronic Obstructive Pulmonary Disease Patients-A Randomized Control Trial. Pak. J. Med. Health Sci..

[39] Liu Y., Ye F., Ma L. (2024). The effect of budesonide combined with bifidobacteria and lactobacilli on lung function and gut microbiota of patients with chronic obstructive pulmonary disease. Pak. J. Med. Sci..

[40] Mao J., Li Y., Bian Q., Xuan Y., Li J., Wang Z., Feng S., Liu X., Tian Y., Li S. (2022). The Bufei Jianpi Formula Improves Mucosal Immune Function by Remodeling Gut Microbiota Through the SCFAs/GPR43/NLRP3 Pathway in Chronic Obstructive Pulmonary Disease Rats. Int. J. Chron. Obstruct. Pulmon. Dis..

[41] Liu Y., Li L., Feng J., Wan B., Tu Q., Cai W., Jin F., Tang G., Rodrigues L.R., Zhang X. (2024). Modulation of chronic obstructive pulmonary disease progression by antioxidant metabolites from *Pediococcus pentosaceus*: Enhancing gut probiotics abundance and the tryptophan-melatonin pathway. Gut Microbes.

[42] Kim H.-j., Lee J.-H., Kwon S.-Y., Kwon Y.J., Park M.-S., Kim K.H., Shin C.H., Joo J.-C., Park S.-J. (2024). Effect of a Combination of *Lactiplantibacillus plantarum* KC3 and *Leonurus japonicus* Extracts in Respiratory Discomfort: A Randomized, Double-Blind, Placebo-Controlled Trial. Nutrients.

[43] Liu P., Hong H., Chen Z., Zhou J., Wang S., Wang C., Li D., Tang C. (2024). Integrative metabolomic and microbiomic profiling: Unveiling the therapeutic mechanisms of Yangyinqingfei Decoction in acute lung injury. Food Biosci..

[44] Sun X., Liu F., Hu B., Zhu L., Yang H., Zheng J., Hu H., Liu H. (2025). N-Acetylchitooligosaccharides Alleviate Pulmonary Inflammation and Modulate Glycerophospholipid Metabolism in Murine Acute Lung Injury. Int. J. Mol. Sci..

[45] Cruickshank-Quinn C., Jacobson S., Hughes G., Powell R., Petrache I., Kechris K., Bowler R., Reisdorph N. (2018). Metabolomics and transcriptomics pathway approach reveals outcome-specific perturbations in COPD. Sci. Rep..

[46] Shimizu Y., Horibata Y., Domae M., Nakamura Y., Yokoyama T., Arai R., Shiobara T., Takemasa A., Koike R., Uchida N. (2025). Dysregulated metabolic pathways of pulmonary fibrosis and the lipids associated with the effects of nintedanib therapy. Respir. Res..

[47] Gai X., Guo C., Zhang L., Zhang L., Abulikemu M., Wang J., Zhou Q., Chen Y., Sun Y., Chang C. (2021). Serum Glycerophospholipid Profile in Acute Exacerbation of Chronic Obstructive Pulmonary Disease. Front. Physiol..

[48] Yu Y., Xie Y., He Y., Zheng Y., Gao Z. (2024). Dysregulated serum glycerophospholipid and fatty acid metabolism in different disease stages of COPD. Eur. Respir. J..

[49] Godbole S., Bowler R.P. (2022). Metabolome Features of COPD: A Scoping Review. Metabolites.

[50] Correnti S., Preianò M., Gamboni F., Stephenson D., Pelaia C., Pelaia G., Savino R., D’Alessandro A., Terracciano R. (2024). An integrated metabo-lipidomics profile of induced sputum for the identification of novel biomarkers in the differential diagnosis of asthma and COPD. J. Transl. Med..

[51] Wu J., Qiao Y., Jin W., Jia F., Wang Z., Li L., Cheng F., Zhao W., Cheng Y., Zhao L. (2024). Metabolomics and 16S rDNA sequencing of intestinal flora reveal the regulation of *Sparassis latifolia* polysaccharides on splenic immune function in lead-exposed mice. Int. J. Biol. Macromol..

[52] Chen M., Yao H., Tan H., Huang W., Wu Q., Nie S. (2023). Impact of *Bifidobacterium longum* NSP001 on DSS-induced colitis in conventional and humanised mice. Food Sci. Hum. Wellness.

[53] Wu T., Sun M., Liu R., Sui W., Zhang J., Yin J., Fang S., Zhu J., Zhang M. (2020). *Bifidobacterium longum* subsp. *longum* Remodeled *Roseburia* and Phosphatidylserine Levels and Ameliorated Intestinal Disorders and liver Metabolic Abnormalities Induced by High-Fat Diet. J. Agric. Food Chem..

[54] Mari P.-V., Carriera L., Saviano A., Ricci A., Coppola A., Ielo S., Lipsi R., Dodaj M., Gennari F., Gullà M. (2026). Impact of IL-4/IL-13 Blockade with Dupilumab on the Microbiome in Type 2 Inflammatory Diseases: A Systematic Review. Curr. Allergy Asthma Rep..

[55] Ciesielska-Markowska I., Mycroft-Rzeszotarska K., Korczyński P., Pulik K., Górska K. (2026). Characteristics of Respiratory Microbiome in COPD—A Literature Review. Adv. Respir. Med..

