# Supplementary material for: Re-Du-Ning Alleviates Airway-Intestinal Inflammation of COPD Rats by Regulating Glycerophospholipid Metabolism and Modulating Gut Microbiome
Source: Med Sci (Basel). 2026 Oct 12;14(6):662. doi: 10.3390/medsci14060662 (PMC13707085; doi:10.3390/medsci14060662)
Supplement: Supplementary file 1 [file medsci-14-00662-s001.zip › medsci-4595026-supplementary.pdf]

Supplementary Data for Re-Du-Ning Alleviates Airway-Intestinal Inflammation of COPD Rats by Regulating Glycerophospholipid Metabolism and Modulating Gut Microbiome

Table S1 Identification of differential metabolites in lung

No.	Metabolites	t_R/min	m/z	HMDB ID	Trends[a]	Trends[b]
1	PS(18:0/18:1(9Z))	14.463	810.53060	HMDB0010163	↓	↑
2	PC(18:0/0:0)	8.228	546.35478	HMDB0010384	↑	↓
3	ADP	1.453	428.03703	HMDB0001341	↑	↓
4	PA(16:0/14:0)	14.616	621.44412	HMDB0114833	↓	↑
5	NADPH	14.64	710.07781	HMDB0000221	↓	↑

Note: Trends[a]: the change trend of the model group compared to the blank group; Trends[b]: the change trend of the RDN group compared to the model group; ↓ means down, ↑ means up

Table S2 Identification of differential metabolites in serum

No.	Metabolites	t_R/min	*m*/*z*	ID	Trends[a]	Trends[b]
1	Zingerone	6.263	193.08672	HMDB32590	↑	↓
2	N-Ethyl trans-2-cis-6-nonadienamide	6.302	199.18081	HMDB32273	↑	↓
3	Deoxycholic acid	6.980	391.28543	HMDB00626	↓	↑
4	Sphinganine 1-phosphate	7.199	382.27228	HMDB01383	↑	↓
5	PC(15:0/0:0)	7.563	482.32497	LMGP01050016	↓	↑
6	Sphingosine-1-phosphocholine	7.666	482.36978	LMSP01060001	↓	↑
7	PC(20:4(5Z,8Z,11Z,14Z)/0:0)	7.741	544.34058	LMGP01050048	↓	↑
8	PC(14:0/0:0)	8.685	466.29453	LMGP01050012	↑	↑
9	CDP-glucose	8.818	588.06491	HMDB03369	↓	↑
10	PA(18:2(9Z,12Z)/0:0)	8.862	433.23560	LMGP10050017	↓	↑
11	Etiocholanolone glucuronide	8.913	501.22320	HMDB04484	↓	↑
12	PS(22:4(7Z,10Z,13Z,16Z)/15:0)	8.943	815.55352	LMGP03010791	↓	↑
13	PA(21:0/17:0)	8.998	771.52750	LMGP10010681	↓	↑
14	NAD	10.106	647.11029	HMDB00902	↓	↑

Note: Trends[a]: the change trend of the model group compared to the blank group; Trends[b]: the change trend of the RDN group compared to the model group; ↓ means down, ↑ means up

Table S3 Identification of differential metabolites in feces

No.	Metabolites	tR/min	m/z	ID	Trends[a]	Trends[b]
1	Monomenthyl succinate	1.409	277.14055	HMDB36143	↑	↓
2	3-Dehydroxycarnitine	1.457	146.11794	HMDB06831	↑	↓
3	Glyceryl 5-hydroxydecanoate	2.420	280.21279	HMDB32297	↓	↑
4	2-amino-8-oxo-9,10-epoxy-decanoic acid	2.652	238.10655	LMFA01060176	↓	↑
5	1H-Indole-3-carboxaldehyde	3.553	146.06041	HMDB29737	↓	↑
6	PC(14:0/0:0)	4.055	468.30823	LMGP01050012	↑	↓
7	Menthyl pyrrolidone carboxylate	4.491	341.19498	HMDB32368	↑	↓
8	Dihydrocortisol	4.583	363.21766	HMDB03259	↑	↓
9	Frangulanine	4.908	481.31700	HMDB30199	↑	↓
10	xi-8-Hydroxyhexadecanedioic acid	5.523	301.20144	HMDB37831	↑	↓
11	Cholic acid	5.906	426.32237	HMDB00619	↑	↓
12	Hexadecanedioic acid	5.947	285.20689	HMDB00672	↓	↑
13	Lucidenic acid M	6.273	480.33250	HMDB35973	↑	↓
14	Deoxycholic acid	6.341	375.28967	HMDB00626	↑	↓
15	Cervonoyl ethanolamide	6.341	355.26367	HMDB13627	↑	↓
16	7-Ketodeoxycholic acid	6.374	407.27994	HMDB00391	↑	↓
17	TetracoRDNhexaenoic acid	6.507	357.27905	HMDB02007	↑	↓
18	Brassinolide	6.507	445.33178	HMDB34081	↑	↓
19	Deoxycholic acid glycine conjugate	6.569	448.30581	HMDB00631	↑	↓
20	DG(20:2/22:5/0:0)	6.862	729.52789	LMGL02010263	↑	↓
21	Trihydroxy-20-oxo-5b-pregnan-18-al	6.895	363.22068	HMDB06753	↑	↓
22	Chenodeoxycholic acid	6.992	391.28504	HMDB00518	↑	↓
23	13S-hydroxyoctadecadienoic acid	7.308	297.24281	HMDB04667	↑	↓
24	12,13-DHOME	7.308	337.23537	HMDB04705	↑	↓
25	27-Deoxy-5b-cyprinol	7.353	457.33208	HMDB01231	↑	↓
26	Tetrahydrocortisol	7.418	365.23629	HMDB00949	↑	↓

Continued Table S3 Identification of differential metabolites in feces

No.	Metabolites	tR/min	m/z	ID	Trends[a]	Trends[b]
27	N-Oleoyl Dopamine	7.817	418.33178	LMFA08020140	↓	↑
28	Coprocholic acid	7.850	468.36937	HMDB00601	↑	↑
29	7 alpha-Hydroxy-3-oxo-4-cholestenoate	7.984	448.34286	HMDB12458	↑	↑
30	Withanolide B	8.445	455.27829	HMDB30020	↓	↑
31	(3beta,17alpha,23S,24S)-17,23-Epoxy-3,24,29-trihydroxy-27-norlanost-8-en-15-one	8.944	497.32494	HMDB35144	↓	↑
32	13'-carboxy-alpha-tocopherol	9.342	483.34524	HMDB12555	↓	↑
33	SM(d18:1/24:1(15Z))	9.374	813.68570	LMSP03010007	↑	↓
34	8,11,14-EicoRDNtrienoic acid	9.514	324.29004	HMDB02925	↓	↑
35	12,13-dihydroxy-11-methoxy-9-octadecenoic acid	9.789	365.23058	LMFA01080006	↑	↓
36	16(R)-HETE	9.799	343.22235	HMDB04680	↑	↓
37	Ricinoleic acid	9.799	299.25847	HMDB34297	↑	↓
38	6,10,14-Trimethyl-5,9,13-pentadecatrien-2-one	9.979	280.26385	HMDB34495	↑	↓
39	Sorbitan stearate	10.140	467.27766	HMDB29888	↓	↑
40	Lanosterin	10.658	427.39027	HMDB01251	↓	↑

Note: Trends[a]: the change trend of the model group compared to the blank group; Trends[b]: the change trend of the RDN group compared to the model group; ↓ means down, ↑ means up
